# Muscle synergy analysis yields an efficient and physiologically relevant method of assessing stroke

**DOI:** 10.1093/braincomms/fcac200

**Published:** 2022-08-09

**Authors:** Tetsuro Funato, Noriaki Hattori, Arito Yozu, Qi An, Tomomichi Oya, Shouhei Shirafuji, Akihiro Jino, Kyoichi Miura, Giovanni Martino, Denise Berger, Ichiro Miyai, Jun Ota, Yury Ivanenko, Andrea d’Avella, Kazuhiko Seki

**Affiliations:** Department of Mechanical Engineering and Intelligent Systems, The University of Electro-communications, Tokyo 182-8585, Japan; Neurorehabilitation Research Institute, Morinomiya Hospital, Osaka 536-0025, Japan; Department of Rehabilitation, University of Toyama, Toyama 930-0194, Japan; Center for Medical Sciences, Ibaraki Prefectural University of Health Sciences, Ibaraki 300-0394, Japan; Department of Precision Engineering, School of Engineering, The University of Tokyo, Tokyo 113-8656, Japan; Department of Precision Engineering, School of Engineering, The University of Tokyo, Tokyo 113-8656, Japan; Department of Advanced Information Technology, Kyushu University, Fukuoka 819-0395, Japan; Department of Neurophysiology, National Institute of Neuroscience, National Center of Neurology and Psychiatry, Tokyo 187-8502, Japan; Research into Artifacts, Center for Engineering (RACE), School of Engineering, The University of Tokyo, Tokyo 113-8656, Japan; Department of Rehabilitation, Morinomiya Hospital, Osaka 536-0025, Japan; Department of Rehabilitation, Morinomiya Hospital, Osaka 536-0025, Japan; Laboratory of Neuromotor Physiology, IRCCS Fondazione Santa Lucia, Rome 00179, Italy; Wallace H. Coulter Department of Biomedical Engineering, Emory University and Georgia Institute of Technology, Atlanta, GA 30322, USA; Laboratory of Neuromotor Physiology, IRCCS Fondazione Santa Lucia, Rome 00179, Italy; Neurorehabilitation Research Institute, Morinomiya Hospital, Osaka 536-0025, Japan; Research into Artifacts, Center for Engineering (RACE), School of Engineering, The University of Tokyo, Tokyo 113-8656, Japan; Laboratory of Neuromotor Physiology, IRCCS Fondazione Santa Lucia, Rome 00179, Italy; Laboratory of Neuromotor Physiology, IRCCS Fondazione Santa Lucia, Rome 00179, Italy; Department of Biomedical and Dental Sciences and Morphofunctional Imaging, University of Messina, Messina 98122, Italy; Department of Neurophysiology, National Institute of Neuroscience, National Center of Neurology and Psychiatry, Tokyo 187-8502, Japan

**Keywords:** Fugl-Meyer assessment, muscle synergy, stroke, rehabilitation

## Abstract

The Fugl-Meyer Assessment is widely used to test motor function in stroke survivors. In the Fugl-Meyer Assessment, stroke survivors perform several movement tasks and clinicians subjectively rate the performance of each task item. The individual task items in the Fugl-Meyer Assessment are selected on the basis of clinical experience, and their physiological relevance has not yet been evaluated. In the present study, we aimed to objectively rate the performance of task items by measuring the muscle activity of 41 muscles from the upper body while stroke survivors and healthy participants performed 37 Fugl-Meyer Assessment upper extremity task items. We used muscle synergy analysis to compare muscle activity between subjects and found that 13 muscle synergies in the healthy participants (which we defined as standard synergies) were able to reconstruct all of the muscle activity in the Fugl-Meyer Assessment. Among the standard synergies, synergies involving the upper arms, forearms and fingers were activated to varying degrees during different task items. In contrast, synergies involving posterior trunk muscles were activated during all tasks, which suggests the importance of posterior trunk muscle synergies throughout all sequences. Furthermore, we noted the inactivation of posterior trunk muscle synergies in stroke survivors with severe but not mild impairments, suggesting that lower trunk stability and the underlying activity of posterior trunk muscle synergies may have a strong influence on stroke severity and recovery. By comparing the synergies of stroke survivors with standard synergies, we also revealed that some synergies in stroke survivors corresponded to merged standard synergies; the merging rate increased with the impairment of stroke survivors. Moreover, the degrees of severity-dependent changes in the merging rate (the merging rate–severity relationship) were different among different task items. This relationship was significant for 26 task items only and not for the other 11 task items. Because muscle synergy analysis evaluates coordinated muscle activities, this different dependency suggests that these 26 task items are appropriate for evaluating muscle coordination and the extent of its impairment in stroke survivors. Overall, we conclude that the Fugl-Meyer Assessment reflects physiological function and muscle coordination impairment and suggest that it could be performed using a subset of the 37 task items.

## Teaser

Funato *et al.* analyzed muscle synergies during the Fugl-Meyer Assessment and report that posterior trunk synergy was active throughout the FMA, while upper arm, forearm and finger synergies were sequentially active. Synergy merging reflected stroke severity to varying degrees with task items, which enabled the selection of effective items.

## Graphical Abstract

**Graphical abstract:**
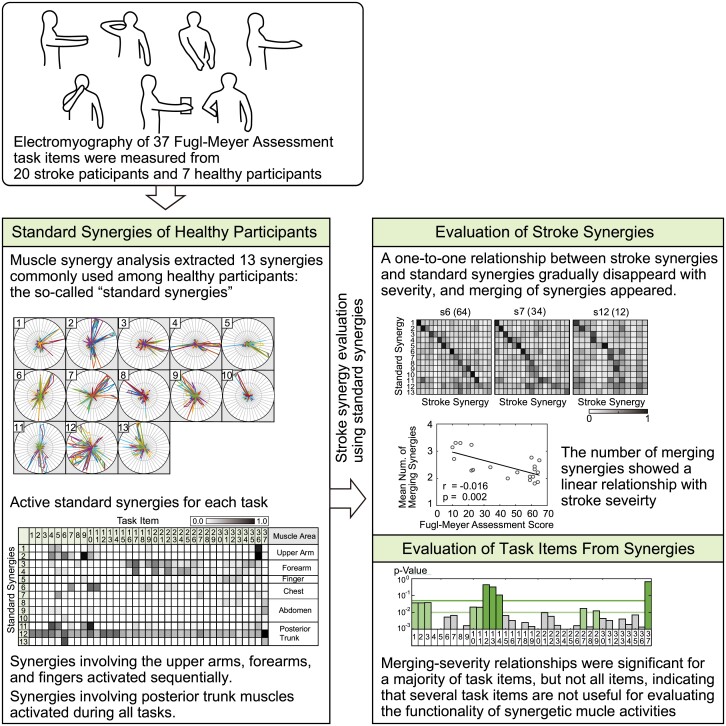

## Introduction

Stroke and traumatic injury to the brain and spinal cord can cause paralysis and reduce movement functionality. Effective clinical interventions require reliable, sensitive and quantitative assessments of patient motor function and recovery. More than 40 years ago, the Fugl-Meyer Assessment (FMA)^1^ was proposed as the first quantitative test for evaluating recovery from sensorimotor impairments. Currently, it is the test most widely used by clinicians for evaluating stroke-related motor impairments. When completing the FMA for upper extremities, stroke survivors are asked to perform actions for 37 items. The items assess simple movements, motor coordination and reflex actions for the shoulder, elbow, forearm, wrist and hand. Clinicians rate patient performance for each task item subjectively and then generate the FMA score by summing the scores for all items.

Although the FMA is well established in terms of its reliability,^2^ validity^3^ and responsiveness,^4^ there is one major critique: each item in the test is chosen on the basis of clinical experiences, but the physiological relevance of the items is never evaluated. Although several attempts have been made to re-evaluate the relevance of the FMA,^5^ most researchers use a subjective approach such as comparing scores generated by experienced and inexperienced clinicians.^6^ Because the implicit assumption regarding the FMA test is that the score represents physiological processes and recovery status of the injured brain,^1^ the test itself should ideally be evaluated using physiological measurements.

In the present study, we used muscle synergy analysis to assess the physiological relevance of the FMA in terms of movement coordination. Groups of muscles temporarily working together, known as muscle synergies, have been proposed as functional building blocks underlying the coordination of complex motor behaviours.^7,8^ In muscle synergy analysis, dimensionality reduction is performed on electromyography (EMG) activities measured simultaneously from many muscles so that temporally consistent muscle patterns are clustered. Muscle activity containing diverse spatiotemporal patterns can be decomposed into muscle synergies, that is, a group of muscles with a specific balance of relative muscle activation as well as a set of specific temporal activation waveforms. Muscle synergy analysis is now widely used to characterize a variety of human motor behaviours, including reaching,^9,10^ posture control^11^ and locomotion.^12,13^ Recent studies have found that the spatiotemporal structure of muscle synergy is flexible.^14,15^ Furthermore, ‘merging’ of synergies has been found in stroke survivors,^16^ with the extent of merging reflecting the degree of residual motor functionality;^17,18^ this finding suggests that muscle synergy analysis in the FMA might also depict recovery from motor coordination impairment. Importantly, relevant neuronal substrates of muscle synergies have recently been proposed in the cortex^19,20^ and downstream regions.^21^ Muscle synergy analysis may therefore be a method to evaluate both stroke severity and recovery from a physiological perspective.

The purpose of the current study was to assess the physiological relevance of the FMA test via muscle synergy analysis. To our knowledge, this study represents the first attempt to validate any clinical scale using muscle synergy analysis. First, we sought to identify the muscle synergies underlying the entire upper extremity FMA test (which includes 37 task items) and re-evaluate each task item according to muscle synergy structures found in healthy participants. Based on the synergy merging–related properties for each item, we selected a subset of the items that may be appropriate for evaluating motor coordination in stroke survivors. Overall, our findings support the physiological relevance of the FMA test but also demonstrate that the number of task items can be reduced when a selective diagnosis of muscle coordination function impairment is required.

## Materials and methods

### Experiment

Twenty stroke survivors (two trials per participant; 40 trials in total) and seven healthy participants (four trials for one participant and three trials for all other participants; 22 trials in total) were recruited (see Supplementary Table 1 for the clinical data of stroke survivors and Supplementary Table 2 for the dates and locations of the experiments). The stroke survivors and healthy participants were age and sex matched. Note that all stroke survivors in this study volunteered to participate and all subjects in both the patient group and the age- and sex-matched control group were entirely male. This is likely because female candidates were reluctant to expose their skin for the placement of multiple EMG electrodes on the upper body. Nevertheless, we assume that there are no differences in muscle synergies between males and females. We determined the number of participants on the basis of previous research^17^ into stroke synergy, where 21 and 10 stroke survivors from two hospitals performed different movement tasks depending on the hospitals. Because our experiment assigned the same tasks for all stroke survivors and each participant repeated the tasks twice, we set the number of participants to be approximately that of the first hospital from the previous study.^17^ In the experiment, each participant performed movements of the upper extremities for 37 task items from the FMA (for the complete list of FMA items, see Supplementary Table 3). All participants were able to complete all task items. We measured EMG activity from 41 muscles in the upper body and trunk (for the complete list of measured muscles, see Supplementary Table 4) using wireless EMG sensors (channels 1–31: Mini Wave Infinity, Cometa, Italy) and wired EMG sensors (channels 32–41: Biolog DL-141, S&ME, Japan). Performance was recorded using a conventional video camera. After the experiment, one experienced neurologist replayed the recorded videos and evaluated the performance of each task item to obtain the FMA score for each participant.

### Muscle synergy analysis and standard synergies

Measured EMG data were preprocessed using digital filters (0.1 Hz cutoff high-pass and 20 Hz cutoff low-pass filters) and rectification (see Preprocessing in Supplementary Methods). Subsequently, EMG data from 37 different task epochs were extracted using the recorded movie of the experiment. Muscle synergies were then extracted using non-negative matrix factorization (NMF) (see Synergy Analysis in Supplementary Methods). The number of muscle synergies was determined so that their variance accounted for (VAF) exceeded 0.8.

To assess the stroke-induced changes in muscle synergies, we first defined a set of standard synergies characteristic of healthy participants. We defined the standard synergies as the synergies most frequently observed in healthy participants and searched for them using a cluster analysis. If one type of synergy (cluster of synergies) was used by more than half of participants (i.e. the cluster included synergies from more than half of participants), the type of synergy was regarded as one of the standard synergies (see Standard Synergies in Supplementary Methods, and see Supplementary Note, Supplementary Fig. 1, and Supplementary Fig. 2 for the effects of small EMG activities). To reduce deviations in movement for each task item, and to improve the robustness of the obtained standard synergies, we recorded EMG data from healthy subjects performing each FMA task item repeatedly (three or four times), and included the data in our analysis to obtain the standard synergy. To understand the role of each synergy, we selected the muscles with relatively high contributions within the muscles assigned for each synergy. To do this, we selected muscles whose average magnitude of activity among participants exceeded 40–60% of maximal activation.

We also assessed the contribution of each standard synergy to the generation of muscle activity in each of the 37 task items. For this purpose, we calculated the temporal coefficient of standard synergies (see Standard Synergies in Supplementary Methods). We separated the time series of each temporal coefficient by 37 task epochs and averaged them. The active synergies in each task item were the synergies whose averaged temporal coefficient exceeded a threshold. We summarized these active synergies as a synergy–task relationship. We also evaluated the standard synergy–task relationship for stroke survivors. We calculated the temporal coefficient of standard synergies for stroke survivors and then obtained the standard synergy–task relationship.

### Evaluation of the synergies of stroke survivors and re-evaluation of FMA items

To evaluate how stroke severity impacts muscle synergies, we compared the synergies of stroke survivors with the standard synergies. At first, the similarity between each synergy from a patient and each standard synergy was calculated as the normalized scalar product (cosine coefficient). Next, the patient synergies were represented as a combination of standard synergies. The average number of standard synergies that represented the synergies of patients with stroke was calculated as the merging rate (see Merging of Synergies in Supplementary Methods).^17^ The merging rates were then compared with stroke severity (FMA score). We summarized this as the merging rate–stroke severity relationship.

The merging rate–stroke severity relationship can vary across task items. Thus, we calculated the merging rate for each task item (see Merging Rate–Severity Relationship of Task Items in Supplementary Methods). We extracted the active synergies in each task item and evaluated the merging rate for only the extracted active synergies. We obtained the merging rate–stroke severity relationship for each task item by comparing the merging rate of each task item with stroke severity.

### Ethical considerations

The experimental protocol was approved by the Institutional Ethics Committee/Institutional Review Board of Morinomiya Hospital (Approval No. 0221) and the Department of Engineering at the University of Tokyo (Approval No. 16–81). All participants received the written experimental protocol and all signed an informed consent form prior to study initiation in accordance with the declaration of Helsinki.

### Statistical analysis

With the exception of specified cases, we evaluated statistical significance using *t*-tests. The relationships between the merging rate and the FMA score were evaluated using robust linear regression with bisquare weighting.^22^ The regression assumption was checked for the equal variance of the residuals, the independence of the residuals and the normal distribution of the residuals using the Breusch–Pagan test, the Durbin–Watson test and the Anderson–Darling test, respectively. We then calculated the regression coefficient and the significance of the incline. We used the Matlab ‘fitlm’ function to compute the robust linear regression. Merging rate of healthy, mild and severe stroke survivors were compared with one-way analysis of variance (ANOVA), and then differences among the three groups were tested using Tukey-Kramer multiple comparison tests. In addition, we used two-way ANOVA to evaluate the effects of body region and participant type on the standard synergy–task relationship. Regarding the sensitivity to parameters, the thresholds for the standard synergy–task relationship, corresponding muscles for synergies, synergy merging and VAF were investigated using variable parameters (see Discussion).

### Data availability

The data that support the findings of this study are available from the corresponding author upon reasonable request. The program code for the analysis is available from https://fma-synergy-programs.funato.jp.

## Results

### Experiments and muscle synergy analysis

**Figure 1:**
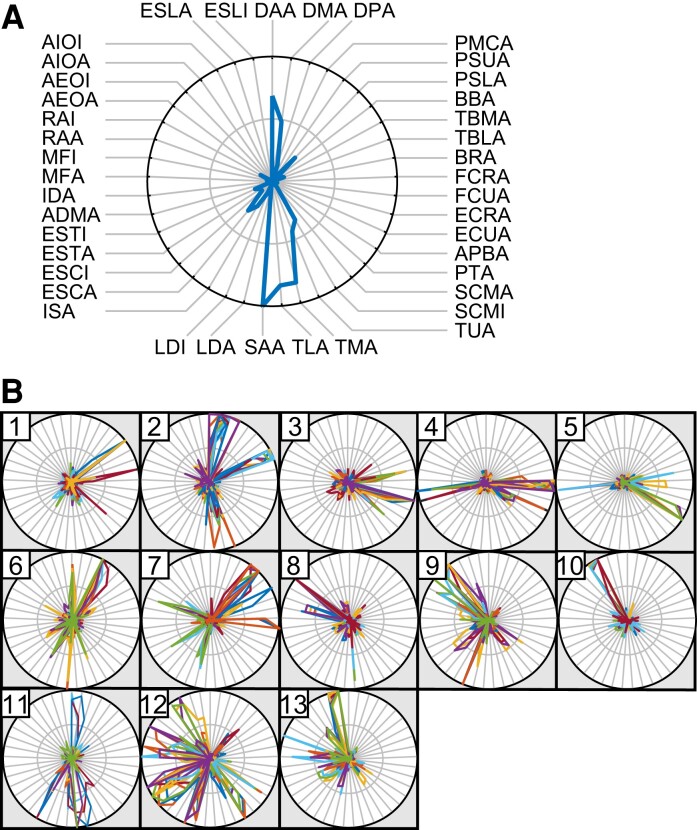
**Muscle synergy analysis.** (**A**) A visualization of the muscle synergies. Muscles are arranged at the node of the circle (see Supplementary Table 4 for the full name of each abbreviated muscle). Hand and finger muscles are arranged mainly at the upper right side, muscles around the arm are at the lower right side, upper trunk muscles are at the lower left side and lower trunk muscles are at the upper left side of the circle. The blue line, which indicates the rate of involvement of the different muscles, shows the muscle synergy. The radius of the circle is set to be the maximum value of synergy. (**B**) Standard synergies obtained as common synergies among healthy participants. Each colour shows one trial from a healthy participant, and synergies with similar patterns are grouped in a circle as a single standard synergy. The numbers at the top left indicate the index of each standard synergy.

We collected a full sequence of FMA items from seven healthy participants (total number of trials = 22) and twenty stroke survivors (total number of trials = 40) and obtained EMG data from all participants (Supplementary Table 3 lists the FMA task items and Supplementary Table 4 lists the measured muscles). The average duration for completing all tasks was 7 min 42 s ± 2 min 18 s (mean ± standard deviation) for healthy participants and 11 min 7 s ± 4 min 27 s for stroke (mild stroke: 9 min 24 s ± 3 min 0 s; severe stroke: 14 min 18 s ± 5 min 2 s). We extracted muscle synergies from EMGs measured in 41 muscles throughout the FMA protocol, including all 37 task items (see Supplementary Fig. 3). Then, we determined the number of synergies for each participant as the minimum number of synergies necessary to adequately reconstruct the EMGs (VAF >0.8, see Methods) for the entire FMA sequence. Overall, the mean numbers of synergies for healthy participants and those with stroke were 16.4 ± 1.2 (mean ± standard deviation) and 16.1 ± 1.9, respectively. When severity of stroke was considered, we found that the number of synergies was smaller in those who had experienced severe stroke (mild stroke: 16.9 ± 1.2; severe stroke: 14.7 ± 2.3, *P* < 0.001, *t* = 3.9, *t*-test). Although the number of synergies did not differ significantly between healthy participants and those with mild stroke (*P* = 0.16, *t* = –1.4, *t*-test), as in previous reports, it was significantly smaller in survivors of severe stroke (*P* = 0.008, *t* = 2.8 versus healthy participants, *t*-test).^16,17^ This indicates that FMA performance in patients who had experienced severe stroke relied on a smaller number of muscle synergies. However, the average number of synergies in stroke participants was close to that of healthy subjects; this finding was different from the results of previous research.^16,17^ The way in which the tasks were given potentially caused this difference. Whereas previous studies used relatively simple tasks, such as reaching,^17^ our experiment imposed a variety of movements in the FMA. FMA task items were more challenging for the stroke patients than for healthy participants, and the stroke patients needed to perform more ineffectual movements to accomplish each task. We therefore consider that the increased number of muscle synergies that stroke patients used to achieve difficult tasks counterbalanced the decreased synergies from merging, thus resulting in a nearly equal number of synergies between the healthy participants and the stroke patients. Next, to evaluate the extracted muscle synergies, we examined how the 41 muscles contributed to each muscle synergy (Fig. 1). An example of the resulting muscle weights for one synergy can be seen in Fig. 1A.

### Standard synergies among healthy participants

We identified 13 ‘standard synergies’ from the healthy participant data (Fig. 1B). These standard synergies represent the synergies commonly observed in the majority of the healthy participants (see Methods). Each standard synergy was then classified based on the characteristics of the muscles that were a significant part of it (Supplementary Table 5). We found that the standard synergies could thus be classified as primarily upper arm (synergy 1 and 2), forearm (3 and 4), finger and thumb (5), chest (6 and 7), abdomen (8–10) and posterior trunk (11–13). This result indicates that the muscle synergies coordinating different parts of the upper body are recruited by the entire sequence of FMA items.

**Figure 2:**
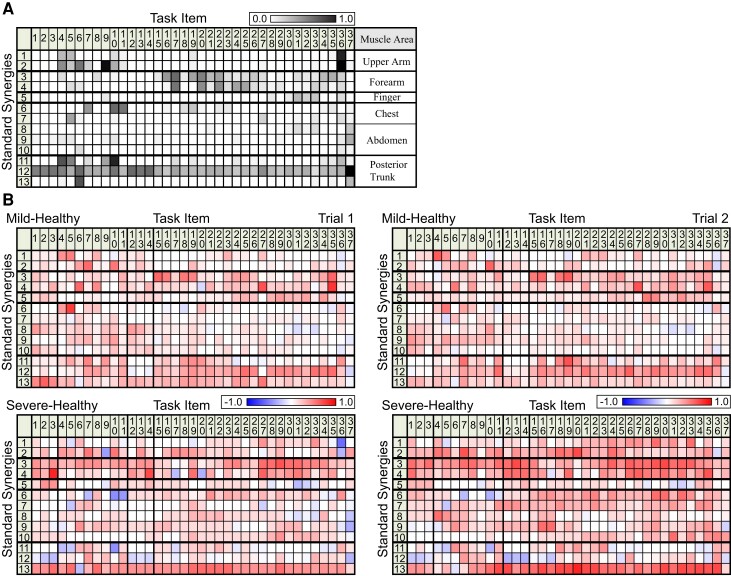
**Standard synergy–task relationship.** (**A**) A standard synergy–task relationship in healthy participants. The synergies observed in each task item are displayed according to the number of active trials normalized by the total number of trials, which was 22. The right-most column, labelled ‘Muscle Area’, displays the corresponding muscle area with synergies (Supplementary Table 5). (**B**) The difference in the standard synergy–task relationship between those with mild/severe stroke (see also Supplementary Fig. 4) and the healthy participants (**A**). Positive values (higher activity in patients than in healthy participants) are shown in red and negative values (lower activity in patients) are shown in blue.

Next, we investigated how these standard synergies were recruited in each of the 37 task items on the FMA test. For each item, we assessed the extent to which each standard synergy contributed to the movements, calculated as the frequency with which it was recruited among the participants (Fig. 2A). We found that each standard synergy was differentially recruited throughout the time course of the full FMA sequence. The synergies of the upper arm (synergy 1 and 2) were primarily recruited early in the FMA sequence (i.e. items 4–10), those of the forearm (synergy 3 and 4) were recruited in the middle of the sequence (i.e. items 16 to 25) and those of the finger and thumb (synergy 5) were used late in the sequence (i.e. items 31 to 36). Synergies of the anterior trunk (synergy 6–10) were recruited sparsely. Interestingly, the synergies involving posterior trunk muscles (synergy 11–13) were recruited for most items, and one of these synergies (synergy 12) was recruited for all of the task items. This result implies that posterior trunk muscle synergies are important throughout the entire FMA sequence, both for performing distal, discrete movements and as part of reflexive movements.

In summary, we found that (i) muscle activity during a complete sequence of FMA items could be characterized by 13 muscle synergies that primarily represent coordination within different parts of the body, (ii) different task items were performed using different combinations of muscle synergies, and (iii) the synergies for posterior trunk muscles were recruited for almost all items. Next, we evaluated how these characteristics were affected by stroke severity.

### Evaluation of stroke-induced changes in muscle activity and synergies

We first used standard synergies from healthy participants to examine how muscle activity differed in stroke survivors. The results can be seen in Fig. 2B (see also Supplementary Fig. 4). Specifically, we examined how frequently stroke survivors recruited each standard synergy when performing each task item. Positive values (red) indicate that the corresponding standard synergy was used more frequently in patients with mild (top) or severe (bottom) symptoms than in healthy controls, and negative values (blue) indicate the standard synergies that were used less frequently. A two-way ANOVA with Participant Type (healthy, mild stroke and severe stroke) and Body Region (arm, anterior trunk and posterior trunk) revealed significant differences in both Participant Type (patient trial 1: *F*_2,2_ = 93.0, *P* < 0.001; patient trial 2: *F*_2,2_ = 169.6, *P* < 0.001) and Body Region (patient trial 1: *F*_2,2_ = 143.3, *P* < 0.001; patient trial 2: *F*_2,2_ = 122.8, *P* < 0.001). A *t*-test comparing mild and severe cases of stroke in terms of individual body areas found significant differences in arm synergies (synergy 1–4, trial 1: *P* = 0.01, *t* = –2.5; trial 2: *P* < 0.001, *t* = –6.1) as well as anterior trunk synergies for trial 2 (synergy 6–10, trial 1: *P* = 0.06, *t* = –1.9; trial 2: *P* < 0.001, *t* = –7.4); in contrast, there were no significant differences in posterior trunk synergies (synergy 11–13, trial 1: *P* = 0.11, *t* = 1.6; trial 2: *P* = 0.79, *t* = 0.3). A *t*-test comparing healthy participants and patients with mild stroke revealed significant differences in synergies for all body areas (*P* < 0.001).

**Figure 3:**
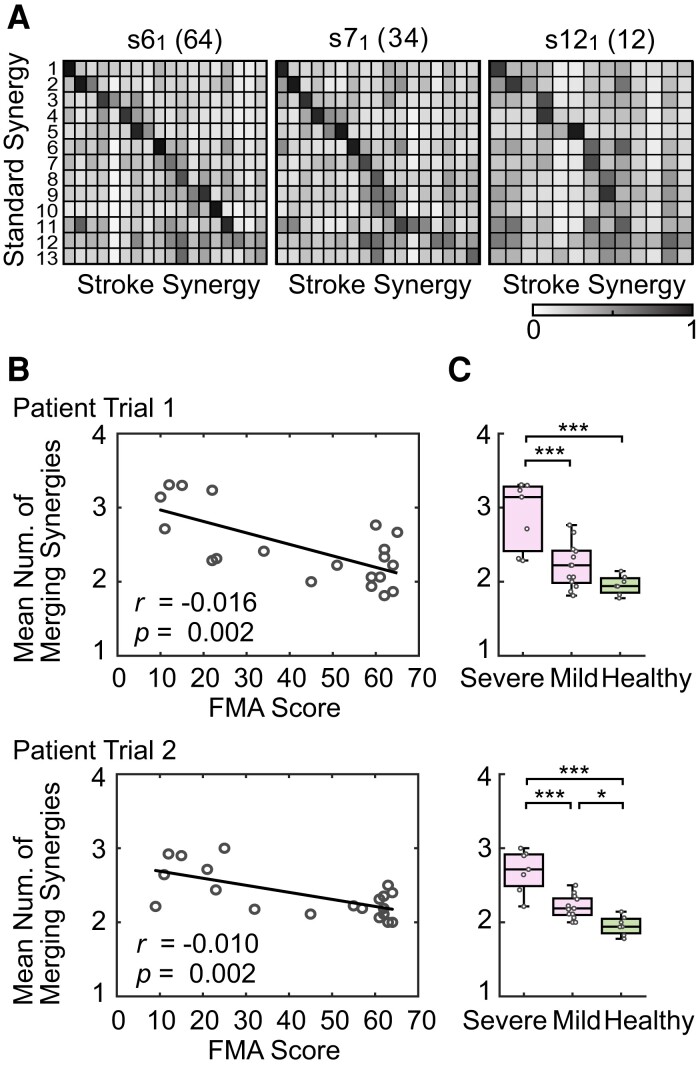
**Relationship between standard synergies and stroke synergies.** (**A**) Correlation of synergies. The rows show the 13 standard synergies and the columns show the stroke synergies. The colours indicate the value of the correlation coefficient between the row (standard synergy) and the column (stroke synergy). The black shade indicates a high value and white indicates a low value. The IDs of each stroke patient are attached to each figure. The subscript of the ID number is the trial number. The numbers next to the IDs are the FMA scores. The results for patients with mild (score: 64), moderate (32) and severe (12) stroke are presented. (**B**) The merging rate for each participant. Each point shows the merging rate for stroke patients in terms of FMA score (*n* = 20). The black line is the linear regression line. The regression coefficient *r* and significance of the incline *P* indicated that the merging rate increased as FMA score decreased (increasing severity of motor deficit). (**C**) Comparison of the merging rate with two stroke severity levels. Patients with severe stroke were those with FMA scores <30 (*n* = 7), and patients with mild stroke were those with FMA scores ≥ 30 *(n* = 13). Healthy data are from one trial of each healthy participant (*n* = 7). *P < 0.05, ***P < 0.001 (Tukey-Kramer multiple comparison test). *P* = 0.001, *F* = 17.0 (trial 1) and *P* < 0.001, *F* = 27.5 (trial 2) in the one-way ANOVA. *P* < 0.001 (trial 1 and trial 2) between severe and mild patients; *P* = 0.208 (trial 1) and *P* = 0.028 (trial 2) between mild patients and healthy participants; *P* < 0.001 (trial 1 and trial 2) between severe patients and healthy participants in the Tukey-Kramer test.

After using the standard synergies to characterize muscle activity in patients with mild or severe stroke (Fig. 2B), we next computed and characterized the muscle synergies of each patient (patient synergy) directly from patient EMG recordings and compared them with the standard synergies (Fig. 3A). The three different similarity matrices shown in the figure represent the correlations between the standard synergies in healthy participants and stroke synergies in a mild (FMA score = 64), moderate (FMA score = 32) and severe (FMA score = 12) stroke survivor, classified according to FMA score (see Supplementary Fig. 5 for detailed data regarding all stroke survivors). The rows of the matrices refer to standard synergies (number of rows = 13) and the columns refer to stroke synergies (number of columns = 13–18). In these matrices, the value of each correlation coefficient is indicated by the grey level, with black reflecting the highest value. The stroke synergies are ordered according to their similarity with respect to the standard synergies such that stroke synergies most strongly correlated with the first standard synergy are placed in the column at the far left. With this arrangement, a perfect match between stroke and standard synergies would result in a black diagonal. By comparing the three similarity matrices, we found that the structure was close to diagonal for the patient with mild symptoms, suggesting a one-to-one relationship between standard synergies and stroke synergies. In contrast, the black diagonal was incomplete in the matrices representing those with moderate or severe stroke, suggesting that the one-to-one relationship was progressively disrupted with increasing stroke severity. Interestingly, we observed a high degree of similarity between individual stroke synergies and multiple standard synergies (i.e. multiple dark entries in one column), suggesting the ‘merging’ of multiple standard synergies in the stroke survivors.^17^

Fig. 3B represents the degree of stroke synergy merging.^17^ The average number of standard synergies used to reconstruct each stroke synergy is displayed as a function of the FMA score. All values were greater than 1, indicating that each stroke synergy could be generated by merging multiple standard synergies. More importantly, the degree of merging (the merging rate) increased with stroke severity (lower FMA score, linear regression coefficient *r* = –0.016 for trial 1 and *r* = –0.010 for trial 2; significance of the incline *P* = 0.002 for trial 1 and *P* = 0.002 for trial 2). Here, the relationship also satisfied the assumption of linear regression (equal variance of the residuals in the Breusch–Pagan test: *P* = 0.58 for trial 1 and *P* = 0.05 for trial 2, independence of the residuals in the Durbin–Watson test: *P* = 0.75 for trial 1 and *P* = 0.49 for trial 2, and normal distribution of the residuals in the Anderson–Darling test: *P* = 0.23 for trial 1 and *P* = 0.84 for trial 2). These results largely confirmed our observations in Fig. 3A. By comparing merging rates among healthy participants and stroke survivors with different severities (FMA score <30: severe, FMA score >30: mild), we found a significant difference between healthy, mild and severe stroke survivors (Fig. 3C; trial 1: *P* < 0.001, *F* = 17.0 and trial 2: *P* < 0.001, *F* = 27.5 in the one-way ANOVA). Tukey-Kramer multiple comparison test results show significant differences between severe and mild patients for both trials (*P* < 0.001 for both trials) and a significant difference between mild patients and healthy participants for trial 2 (trial 1: *P* = 0.208 and trial 2: *P* = 0.028). From these results, we concluded that the merging rate increases with the severity of stroke.

### Re-evaluation of FMA task items from a muscle synergy perspective

**Figure 4:**
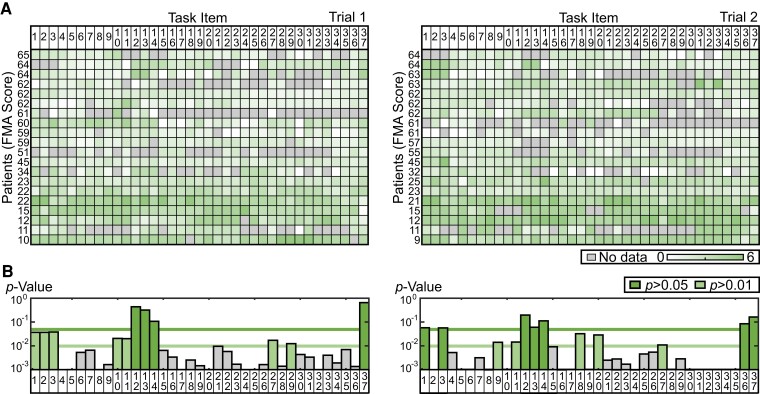
**Task dependency of the severity–merging rate relationship.** (**A**) Averaged merging rate for each patient and each task item. Data in each row show one trial for each patient. Patients are displayed with FMA scores in descending order. Deeper green represents a higher merging rate. Grey represents cases in which the activity of patient synergy for a given task item was too small to evaluate. (**B**) The *P*-values for the linear regression. Each bar represents the results of the linear regression between the FMA score and the merging rate displayed in Fig. A. The two green lines in the *P*-value chart represent *P* = 0.05 and *P* = 0.01. Bars with a green background indicate task items with *P* < 0.05 (dark green) and *P* < 0.01 (light green).

Our data indicate that the severity of stroke is reflected by how much synergy structure deviates from the standard synergy structure (i.e. the extent of synergy merging). Although our data clearly indicated that the FMA reflects underlying physiological processes and recovery, we wanted to determine whether all task items are necessary for arriving at this conclusion. To address this issue, we computed the relative contribution of each task item in determining the degree of synergy merging. Fig. 4A shows the merging rate for each task item. We only evaluated the merging rate for the active synergy of patients in each task. The non-active synergies, which were independent from the standard synergies (i.e. lower than the merging threshold), were handled as no merging rate data. Each element in the matrix in Fig. 4A represents the merging rate for one task item (*columns*) during one FMA trial in one participant (*rows*). Participants are arranged from top to bottom according to the FMA score obtained in each trial. Each column in Fig. 4A therefore represents the relationship between stroke severity and the merging rate observed for each task item. We computed the linear regression between severity and merging rate for each task item (i.e. the relationship in each column). Here, some tasks did not satisfy the assumptions for linear regression. Thus, we removed the data with the largest regression from up to two subjects as outliers, and the assumptions were then satisfied for all tasks (Supplementary Table 6 lists the removed subject data). The resulting linear regression *P*-values are shown in Fig. 4B. The merging rate changed significantly according to stroke severity for some task items (small *P*-values) but not for others (large *P*-values). Specifically, 11 tasks had large *P*-values for patient trial 1 (tasks 12, 13, 14 and 37: *P* > 0.05; tasks 1, 2, 3, 10, 11, 27 and 29: 0.05 > *P* > 0.01). In patient trial 2, 12 tasks had large *P*-values (tasks 1, 3, 12, 13, 14, 36 and 37: *P* > 0.05; tasks 9, 11, 18, 20 and 27: 0.05 > *P* > 0.01). Eight task items overlapped in both trials. Task items with large *P*-values hardly contributed to the evaluation of patient severity in terms of the merging rate. Thus, the ‘merging’ profile of muscle synergies could be represented using a smaller number of task items. Based on the average amount of time required to perform each task item, eliminating the task items that had large *P*-values (trial 1: 11 out of 37; trial 2: 12 out of 37; green bars in Fig. 4B) would reduce the duration of the whole FMA sequence by 22.4% (10 min 46 s versus 8 min 21 s) for trial 1 and 28.4% (11 min 28 s versus 8 min 13 s) for trial 2.

## Discussion

The FMA is one of the most widely used test batteries for assessing motor deficits and recovery in stroke survivors worldwide. In rehabilitation therapy, improving one’s FMA score^1^ is considered to be an important milestone toward motor recovery because it is thought to represent an individual’s level of motor function. However, despite its widespread use, physiological assessments have not been used to test the validity of the FMA in this respect. Thus, information is needed regarding precisely which aspects of motor functionality and recovery are measured, as well as the degree to which the FMA test reflects the extent of damage and recovery in the CNS of stroke survivors. Here, we addressed this issue by considering the processes that underlie coordinated muscle activity, which is crucial for everyday activity. When we evaluated the performance of the entire FMA test battery sequence using muscle synergy analysis, which is an established physiological measure of coordinated muscle activity, we found that (i) muscle activities that comprise the FMA task battery for the upper extremities were composed of 13 different muscle synergies, (ii) each group of synergies had a unique contribution to the performance of each of the 37 FMA task items in all participants, and (iii) the FMA score reflected stroke-related changes in muscle synergy characteristics (synergy merging). These results indicate that the FMA test is a valid tool for assessing the coordinated activity of muscles in the upper arms and fingers as well as stroke-related changes and recovery. We further propose a shorter test battery for measuring muscle coordination in stroke survivors by selecting task items that strongly represent severity-dependent profiles of muscle synergy.

### FMA score represents coordination of muscle activity

As shown in Fig. 2A, muscle coordination throughout the FMA task was constructed from 13 synergies that spanned all regions of the upper body. Specifically, coordination in the upper arm was associated with synergies 1 and 2, the forearm with 3 and 4, the finger and thumb with 5, the chest with 6 and 7, the abdomen with 8–10, and the posterior trunk with 11–13. Notably, many task items preferentially related to one of the upper limb synergies (synergy 1–5). For example, the upper arm synergy (synergy 1 and 2) seemed to underlie the tasks in FMA items 4–10, while the forearm synergy was implicated in items 17 to 27, and synergy of the finger and thumb muscles was related to items 30 to 34. Therefore, we conclude that a majority of the FMA task items are valid tools for evaluating coordinated muscle activities in specific parts of the upper arm. Some task items, however, were insensitive to any upper-arm synergy (for example, items 1–3 and 12–15). All of the insensitive items were meant to evaluate reflex function (Supplementary Table 3). Thus, the FMA also includes task items that are designed for evaluating functions other than muscle coordination in the upper arm.

### Posterior trunk muscle synergy is an important node for recovery from impaired muscle coordination in stroke survivors

In contrast to the aforementioned specificity, we were unable to identify comparable task-item selectivity in the anterior trunk (synergy 7–10) or the posterior trunk (synergy 11–13). The former was inactive for most task items, while some synergies in the latter group (i.e. synergy 12) were active for all task items. As a consequence, the selective recruitment of a given upper arm synergy (synergy 1–5) was always achieved in combination with the recruitment of posterior trunk synergy (synergy 12). We propose that this nonselective pattern of activity in trunk muscle synergy indicates that the recruitment of most upper arm synergies while performing FMA tasks might be associated with the constant recruitment of posterior trunk synergy (synergy 12). This view is likely supported by our observations regarding muscle activities in patients with stroke (Fig. 2; see also Supplementary Fig. 4). Particularly, when compared with healthy subjects, the activity of synergy 12 (posterior trunk) was significantly *increased* in the majority of task items in the patient with mild stroke (see red-toned cells in most ‘synergy 12’ columns in Fig. 2B) but was *decreased* in the patient with severe stroke (blue-toned cells in most ‘synergy 12’ columns in Fig. 2B). These results suggest that, while the patient with mild stroke performed the FMA tasks with elevated activity of synergy 12 (posterior trunk), the patient with severe stroke performed them with lower activity of this posterior trunk synergy. In contrast, the other arm and finger synergies (i.e. synergy 1–7) had increased activity in both mild and severe patients. We therefore suggest that posterior trunk synergy activity might be a key feature for dissociating mild and severe patients and may thus cause the lower FMA scores of severe patients. While mild patients were able to activate the posterior trunk synergy together with arm and finger synergies, severe patients may have lost this coupling between axial and upper limb synergies and were thus forced to perform the task using lower trunk synergy activation. This uncoupling might be a hallmark of severe patients. Woodbury *et al.*^23^ reported that people categorized as having severe impairment were only able to perform distal, single-joint movement, whereas those with moderate impairment were also able to perform proximal, multi-joint movement. It is clear that trunk stability is more crucial for proximal and multi-joint movement than for distal movement using the fingers. It is therefore possible that their patients with severe impairment lost posterior trunk muscle activity, similar to the patients in the present study. This view is also supported by a previous report indicating that an intervention to increase trunk stability facilitated functional recovery of reaching^24,25^ and reach to grasp^26^ movements during stroke rehabilitation.

### How does the FMA test represent the functionality of the CNS?

We recently reported that spatiotemporal patterns of hand muscle synergies are represented by spinal premotor interneurons in non-human primates.^21^ We found that while spatial properties of muscle synergies were represented in the activity of individual spinal premotor-interneurons, temporal activity was not (see Cheung and Seki^27^ for a review). Thus, the temporal activity of muscle synergy might be regulated upstream in the CNS, e.g. by the descending motor tract.^28^ Assuming that the fundamental circuit of the spinal cord is not affected by stroke, including premotor-interneurons that may code for spatial synergies, impaired muscle activity coordination in patients with stroke could be attributed to an impaired ability to generate/assign temporal activity for each spatial synergy. For example, the increased merging rate observed in patients with severe stroke (Fig. 3) might reflect the degree to which descending motor tracts responsible for generating the temporal activity of a given muscle synergy were affected by a stroke. Consequently, the affected spatial synergy (represented by spinal interneurons) may have been activated by a compensatory drive from a source that was originally responsible for the temporal activity of other spatial synergies. We suggest that the lower FMA scores observed in patients with stroke reflect the extent of damage in the region of the CNS responsible for activating spatial synergy that is predominantly represented in the spinal cord. Furthermore, increases in FMA scores after rehabilitation therapy might reflect the degree to which each patient is able to recruit those compensatory mechanisms^29–31^ to improve coordination of muscle activity. Future studies may elucidate the areas in the CNS that are responsible for both synergy activation and recruited compensation by directly measuring them and relating them to the results of muscle synergy analyses.

As discussed, coordinated activity in trunk muscles, especially posterior trunk synergy (synergy 11–13), could be a key element in improving FMA performance, irrespective of the differences between the task items. Two descending motor tracts, the vestibulospinal and reticulospinal tracts, are known to coordinate activity in trunk muscles.^32–34^ During limb movement, the vestibulospinal tract maintains postural equilibrium while the reticulospinal tract regulates postural muscle tone.^34^ Because their axons are known to terminate in multiple segments of the intermediate layer of grey matter in the spinal cord, neurons in these tracts might be involved in generating activity associated with trunk-muscle synergies. Further, the corticobulbar projection, which regulates the activity of the two descending pathways, might be more sensitive to stroke than is the corticospinal tract, which regulates limb-muscle synergies. Thus, recovery of the vestibulospinal and reticulospinal tracts might be critical in improving upper limb functionality and thus FMA score. Recent experiments using an animal model of brain injury have indicated that enhanced cortical projections to subcortical descending tracts are associated with the recovery of arm/hand function.^29–31^

### FMA sub-task items for evaluating coordinated muscle activity in the upper extremities

The analysis shown in Fig. 2 indicates that the FMA is a comprehensive test that can be used to evaluate the activity of muscle synergies in different parts of the upper extremities. Our data also indicate that the FMA is appropriate for assessing stroke-related impairments of muscle coordination because it differentiated muscle coordination among the participant group with sufficient sensitivity (Fig. 3). Although our analysis further revealed that some of the task items may not be appropriate for evaluating muscle coordination in the upper extremities, these items may be sufficiently sensitive to assess other motor functions (e.g. reflexes) (Fig. 4). Further research is necessary to address this issue. Taken together, our data indicate that the FMA is a valid test for comprehensively evaluating motor functionality in the upper extremities.

The comprehensiveness of the FMA enabled us to identify task items that were less correlated with deficits of muscle coordination and to propose a battery of sub-task items for selectively assessing muscle coordination in stroke patients. The proposed task battery consists of 25 or 26 task items. Because all of these task items were selected from the FMA test, which is already familiar to most clinicians, it should be easy to apply in a clinical setting. Fewer task items, and thus a shorter testing duration than the full FMA test, might help to minimize the effort required by both clinicians and patients. The proposed test battery would be particularly advantageous in situations that require the quick assessment of muscle coordination, even if it compromises the comprehensiveness of the FMA.

A number of clinically established test batteries are used to assess motor functionality (e.g. the Box and Block Test^35^ and Action Research Arm Test^36^ to evaluate upper limb function, the Barthel Index^37^ to evaluate activities of daily living and the Stroke Impairment Assessment Set^38^ and The Brunnstrom Approach^39^ to evaluate impairment). Because most of these tests have been designed and refined using mainly clinical knowledge about a target medical condition, direct quantitative assessments of their physiological relevance, like those conducted in this study, are likely to be beneficial. Such assessments may enhance an understanding of the clinical symptoms from a physiological perspective and also facilitate the future development of novel test batteries with higher physiological relevance.

Several previous attempts have been made to reduce the set of task items and thus generate a shorter version of the FMA. For example, Hsieh *et al.*^40^ selected six FMA items for assessing the upper extremities based on a psychometric analysis of a large pool of FMA test data. They confirmed the validity of their test by comparing scores with those on the full FMA. Importantly, their approach was different from ours because the aim of the present study was not simply to make a shorter version of the FMA test but to propose a test battery that could efficiently measure muscle coordination functionality in stroke survivors with a smaller set of task items. Nevertheless, we found that our new test battery included all six of the task items selected by Hsieh *et al.*^40^ Therefore, we hope that our test battery has significantly enhanced sensitivity to coordinated muscle activity at the individual level compared with the previous short version of the FMA test.^41^

### Parameter sensitivity

This research used several fixed parameters in the analysis; thus, we examined how each parameter affected the results. When we changed the thresholds of the standard synergy–task relationship for healthy subjects to 0.1, 0.2, 0.4 and 0.5 (Supplementary Fig. 6A), the number of active standard synergies for each task item increased with lower thresholds and decreased with higher thresholds. However, regardless of the threshold, synergy 12 (posterior trunk) was still active across most task items, and synergies were sequentially active starting with synergies related to the upper limbs. We also tested the effects of the threshold that was used to examine the relationship between each standard synergy and muscles, by changing the threshold to 0.4, 0.5 and 0.6 (Supplementary Table 5). Although the standard synergy included more muscles when a lower threshold was used, the newly added muscles were mostly from the same anatomical group. This result indicates that the association between each standard synergy and the muscle activity in each body part hardly changes with differing thresholds.

Next, we examined the effects of thresholds on merging in the merging rate–stroke severity relationship in stroke patients. When the threshold for merging (0.1) was varied from 0.08 to 0.12, there was a larger number of merging synergies for patients with smaller FMA scores (Supplementary Fig. 6B) irrespective of thresholds. The same analysis revealed a lower number of merging synergies with a higher threshold, which caused an overall increase in *P*-values for the merging rate–stroke severity relationship (Supplementary Fig. 6C). Nonetheless, the changes in *P*-values with the changes in threshold were smaller than the differences in *P*-values between task items, thus confirming that the merging rate–stroke severity relationship had a certain robustness with respect to the threshold for merging.

Finally, we tested the influence of higher VAF thresholds (0.8, 0.85 and 0.9) on each of the results that was presented (Supplementary Fig. 7). We confirmed an increased number of synergies [0.8: 16.4 ± 1.2 (see Fig. 1), 0.85: 19.7 ± 1.0, 0.9: 24.0 ± 1.2] and standard synergies (13 for 0.8, 19 for 0.85 and 25 for 0.9) with higher thresholds, as expected. We also noted that some of the standard synergies that were defined using the 0.8 threshold were divided into multiple standard synergies using the 0.85 and 0.9 thresholds (Supplementary Fig. 7A). Nevertheless, both the consistent involvement of posterior trunk synergy (synergy 12) throughout the FMA and the link between each task item and responsible standard synergy seemed to be unchanged using higher VAF thresholds. Furthermore, the relationship between the number of merging synergies and severity was unchanged when larger VAF thresholds were used (Supplementary Fig. 7B). Although the results of the merging rate–stroke severity relationship for each task item showed an overall trend toward larger *P*-values when the VAF thresholds were larger than 0.8 (Supplementary Fig. 7C), the task items with larger *P*-values remained unchanged.

## Supplementary Material

fcac200_Supplementary_Data

## Abbreviations

FMA = =: Fugl-Meyer assessment
NMF = =: non-negative matrix factorization
VAF = =: variance accounted for
