# Supplementary material for: Muscle synergy analysis yields an efficient and physiologically relevant method of assessing stroke
Source: Brain Commun. 2022 Aug 9;4(4):fcac200. doi: 10.1093/braincomms/fcac200 (PMC9374474; doi:10.1093/braincomms/fcac200)
Supplement: fcac200_Supplementary_Data [file fcac200_supplementary_data.docx]

# Supplementary Information for

Muscle synergy analysis yields an efficient and physiologically relevant way of assessing stroke

Tetsuro Funato, Noriaki Hattori^†^, Arito Yozu^†^, Qi An, Tomomichi Oya, Shouhei Shirafuji, Akihiro Jino, Kyoichi Miura, Giovanni Martino, Denise Berger, Ichiro Miyai, Jun Ota, Yury Ivanenko, Andrea d'Avella, and Kazuhiko Seki

^†^Noriaki Hattori and Arito Yozu contributed equally to this work

Kazuhiko Seki

Email: seki@ncnp.go.jp

**This PDF file includes:**

Supplementary Methods

Supplementary Note

Supplementary Figures 1 to 7

Supplementary Tables 1 to 6

Supplementary References

# Supplementary Methods

**Preprocessing.** Digitized EMG data (at 2000 Hz) were first down-sampled to 100 Hz. The data were then filtered using a high-pass filter (0.1-Hz cutoff frequency), rectified, and filtered using a low-pass filter (20-Hz cutoff frequency, 4th order Butterworth filter). Subsequently, EMG data were segmented into 37 different task epochs. For this purpose, the clinician who administered the FMA to the patients explicitly recorded the start and end time of each task on the audio track of the video by saying the words "start" and "stop". We used the onset of each word for this segmentation, and data recorded between each task epoch were eliminated. The amplitude of the EMG for each muscle was then normalized by averaging the muscle activity. EMG activity for each muscle was divided by the net EMG activity for the entire task (see also Supplementary Note for the effects of normalization and of small EMG signals).

**Synergy Analysis.** Muscle synergies were identified using non-negative matrix factorization (NMF) with the multiplicative update method.^1^ If a matrix *M* is arranged so that each column is the time series of the EMG activity for each muscle, NMF decomposes this matrix *M* as the product of two matrices *C* and *W*:

$$\begin{aligned} M=CW^{T}+re=\sum c_{i}w_{i}^{T}+re,\#\left( 1 \right) \end{aligned}$$

where the vectors *w_i_*, columns of matrix *W*, are muscle synergies; the vectors *c_i_*, columns of matrix *C*, are their temporal coefficients; and '*re*' represents the residuals. EMG data, represented as *M,* only have non-negative values. Thus, NMF decomposes the matrix so that the decomposed matrices *C* and *W* also have only non-negative values. We used the Matlab "nnmf" function to compute NMF. We used a multiplicative update algorithm for the factorization, and the tolerance for change in the norm of the $re$ matrix ("TolFun" parameter in the “nnmf” function) was 10^−6^. The number of synergies was determined by setting a threshold on the variance accounted for (VAF). To clarify the effect of severity, we determined the number of synergies for each participant, assuming that the trial-by-trial variation in synergies within the same participant was small. We calculated the VAF for each trial and selected the minimum number of synergies with VAF > 0.8 as the number of synergies for that trial. Then, we used the minimum number of synergies among all trials for the same participant as the number of synergies for that participant. For potential differences and similarities with other methods used to extract muscle synergy, see earlier works.^2, 3^

**Standard Synergies.** To extract the standard synergies, we performed a cluster analysis on all synergies from all healthy participants. In the cluster analysis, we evaluated similarity (distance) among synergies using the cosine distance, and we searched for groups of synergies with similarity values exceeding 0.8. If one type of synergy (cluster of synergies) was used by more than half of participants (i.e., the cluster included synergies from more than half of participants), the type of synergy was regarded as one of the standard synergies. We used the Matlab "pdist" function and the "linkage" function to compute the distance, and the “cluster” function to perform clustering.

The synergy-task relationship was estimated according to the following steps: (1) the temporal coefficients of the standard synergies were calculated, (2) the temporal coefficients in each task item were averaged to quantify the activation of each synergy in each task, and (3) steps 1 and 2 were repeated for every trial and the numbers of synergies activated in each trial were counted.

The temporal coefficients of the standard synergies *C^h^* were calculated as follows. From Eq. (1), the muscle activity in each healthy participant *M^h^*, standard synergy *W^h^*, and temporal coefficient *C^h^* had the following relationship:

$$\begin{aligned} M^{h}=C^{h}{W^{h}}^{T}+re\#\left( 2 \right) \end{aligned}$$

To calculate *C^h^*, muscle activity *M^h^* was decomposed with a fixed *W^h^* matrix. The decomposition algorithm was based on the multiplicative update algorithm for NMF. The initial values of *C^h^* were set using a random matrix, and *C^h^* was updated according to the following rule:

$$\begin{aligned} {C_{i}^{h}}^{+}\leftarrow C_{i}^{h}\frac{\left( {C^{h}}^{T}M^{h} \right)_{i}}{\left( {C^{h}}^{T}C^{h}{W^{h}}^{T} \right)_{i}}\#\left( 3 \right) \end{aligned}$$

This operation was repeated until the amount of change in *C^h^* induced by one iteration was lower than a certain value (10^−12^). In this way, *C^h^* was determined and then used as a temporal coefficient.

From the resulting temporal coefficient, we were able to identify the tasks that used each synergy by evaluating the activity of the synergies in each task. For this purpose, we separated the time series of each temporal coefficient according to the 37 tasks and averaged the activity in each task. This operation produced one averaged activity value for each task and each synergy. Tasks for which the averaged activity exceeded a certain value were considered to be tasks that corresponded to the synergies. In the current study, this threshold was set at 0.3. We then obtained the relationships between the standard synergies and tasks for each trial. We summarized the standard synergy-task relationships for all trials and all participants, and then investigated the role of standard synergies in the FMA tasks.

We also evaluated the standard synergy-task relationship for stroke survivors. We calculated the temporal coefficient of standard synergies for stroke survivors *C^s^* from eq. (2) and eq. (3) using the muscle activity of each stroke patient *M^s^* and standard synergy *W^h^*. We then obtained the standard synergy-task relationship via the same method used to calculate that for the healthy participants.

**Merging of Synergies.** The similarity between each patient’s synergy (stroke synergy) and each standard synergy was calculated as the normalized scalar product (cosine coefficient). If the number of standard synergies was *n^h^* and the number of stroke synergies was *n^s^*, the table of cosine coefficients became a *n^h^* × *n^s^* matrix. For each participant and FMA sequence, the columns of this table were re-ordered according to the best match between standard synergies and those in the patients. More specifically, the rows with the maximum cosine coefficient were first identified in each column. Then, we sorted the columns of the table according to the rows with the highest cosine coefficients. The way in which the data in this table varied according to stroke severity indicated the process of divergence from standard synergies.

Previous studies have shown that synergies in the affected arm of a stroke survivor can be characterized by merging multiple synergies identified in the unaffected arm.^4^ The degree of merging can be calculated using a merging coefficient *m_k_^i^* defined as follows:

$$\begin{aligned} w_{i}^{s}\cong\sum_{k=1}^{n^{h}} m_{k}^{i}w_{k}^{h}\#\left( 4 \right) \end{aligned}$$

$$m_{k}^{i}\geq0, i=1\ldots n^{s}$$

Here, *w_i_^s^* and *w_k_^h^* are the *i*-th stroke synergy and the *k*-th standard synergy, respectively. In Eq. (4), the stroke synergy *w_i_^s^* is represented by a linear sum of standard synergies *w_k_^h^*, and the merging coefficient *m_k_^i^* is the nonnegative coefficient of this linear sum. If the merging coefficient *m_k_^i^* exceeds a threshold, this indicates that the standard synergy *w_k_^h^* contributes to the synergy *w_i_^s^*. In the present study, this threshold was set to 0.1. The number of standard synergies that contribute to the *n^s^* stroke synergies can be derived, and the average merging rate could be calculated by dividing this number by *n^s^*. We used the nonnegative least squares method, implemented as the "lsqnonneg" function in Matlab, to calculate the merging coefficient *m_k_^i^*.

**Merging rate-Severity Relationship of Task Items.** To determine the difference in the merging rate-stroke severity relationship between task items, we calculated the merging rate for each task item. A different combination of synergies contributes to each task item. Thus, by extracting the active synergies in one task item and evaluating the merging rate only for extracted active synergies, we were able to estimate the merging rate of that task item. Moreover, this combination of active synergies could be calculated from the temporal coefficient of each synergy. Therefore, the merging rate for each task item could be obtained according to the following three steps: (1) The temporal coefficients for each patient were separated and averaged by task, and the number of synergies for which the averaged activation exceeded a certain value during that task item were counted. The threshold was set to 0.3. (2) The combinations of synergies extracted in one task were compared with the standard synergies, and the averaged merging rate for the task was calculated. (3) From the resulting merging rate for each synergy, the relationship between stroke severity and merging rate was evaluated for each task. The linear regression between stroke severity (FMA score) and merging rate was calculated, and the relationship was evaluated using the linear regression coefficient and *p*-values.

# Supplementary Note

The results of the synergy analysis indicated that EMG activity recorded from healthy participants during the FMA could be reconstructed using 13 standard synergies corresponding to different body areas. These synergies were active in different task items, and the trunk synergies were active in the majority of the task items. The FMA focuses on assessments of upper limb motion and the trunk muscles that mainly support limb motion. Thus, the EMG activity of the trunk muscles was generally small and the signal-to-noise ratio was low. Thus, it is possible that the synergies extracted from the small EMG activity of trunk muscles could be dominated by noise. To clarify the effect of small EMG activity, we analyzed the standard synergies of healthy participants after eliminating the small EMG activity using the following two methods: (1) eliminating small EMG signals, and (2) eliminating the muscles with small EMG signals.

To evaluate the effect of eliminating the small EMG signals, EMG activities for which the absolute value was lower than a given threshold were set to zero. We tested both a 10 μV threshold and a 20 μV threshold. Then, we calculated the VAF of the healthy participants, individual synergies, and the standard synergies of healthy participants. When we set the threshold of the VAF at 77% for the 10 μV condition and 74% for the 20 μV condition, we obtained the same number of standard synergies as in the original analysis. Supplementary Fig. 1 shows these results. In the case of the 10 μV threshold, the cosine correlation for 9 out of 13 synergies exceeded 0.9. In the case of the 20 μV threshold, the cosine correlation for 10 out of 13 synergies exceeded 0.7. In particular, the correlation of trunk synergies (synergies 11, 12, and 13) exceeded 0.8 in both cases. Moreover, the standard synergy-task relationship showed that the trunk synergies were active for the majority of the task items.

We further analyzed the effect of small EMG signals by eliminating the muscles that produced them. For this purpose, we calculated the average rectified EMG activity for each muscle in each trial. If the average rectified EMG activity of a muscle was lower than a given threshold (5 μV or 7 μV), the activity of that muscle in that trial was set to zero for the whole sequence. Supplementary Fig. 2A shows the eliminated muscles for each trial. For the 5-μV threshold, activity of 4.4 muscles on average was set to zero for the whole sequence. For the 7-μV threshold, activity of 7.0 muscles on average was set to zero. The threshold of the VAF was set to 82% for both conditions to match the number of standard synergies selected in the original analysis. Supplementary Fig. 2B, C shows the results for the standard synergies. For the 5-μV threshold, the cosine correlation for 9 out of 12 synergies exceeded 0.9. For the 7-μV threshold, the cosine correlation for 7 out of 12 synergies exceeded 0.9. In particular, the correlation of two out of three trunk synergies (synergy 11 and synergy 12) exceeded 0.9. The characteristics of the trunk synergies that were active for the majority of the task items were typical for synergy 11 and synergy 12, indicating that the similarity of these two synergies was important. Moreover, the standard synergy-task relationship (Supplementary Fig. 2C) indicated that the trunk synergies remained active for the majority of the task items.

The standard synergies were generally robust given the elimination of small EMG activity. In particular, the trunk synergies (synergy 11 and 12) were hardly affected by the elimination. Therefore, the characteristics of trunk synergies are not considered to be by-products of statistical analyses in which EMG activity is small, but instead to be real characteristics of trunk activity.


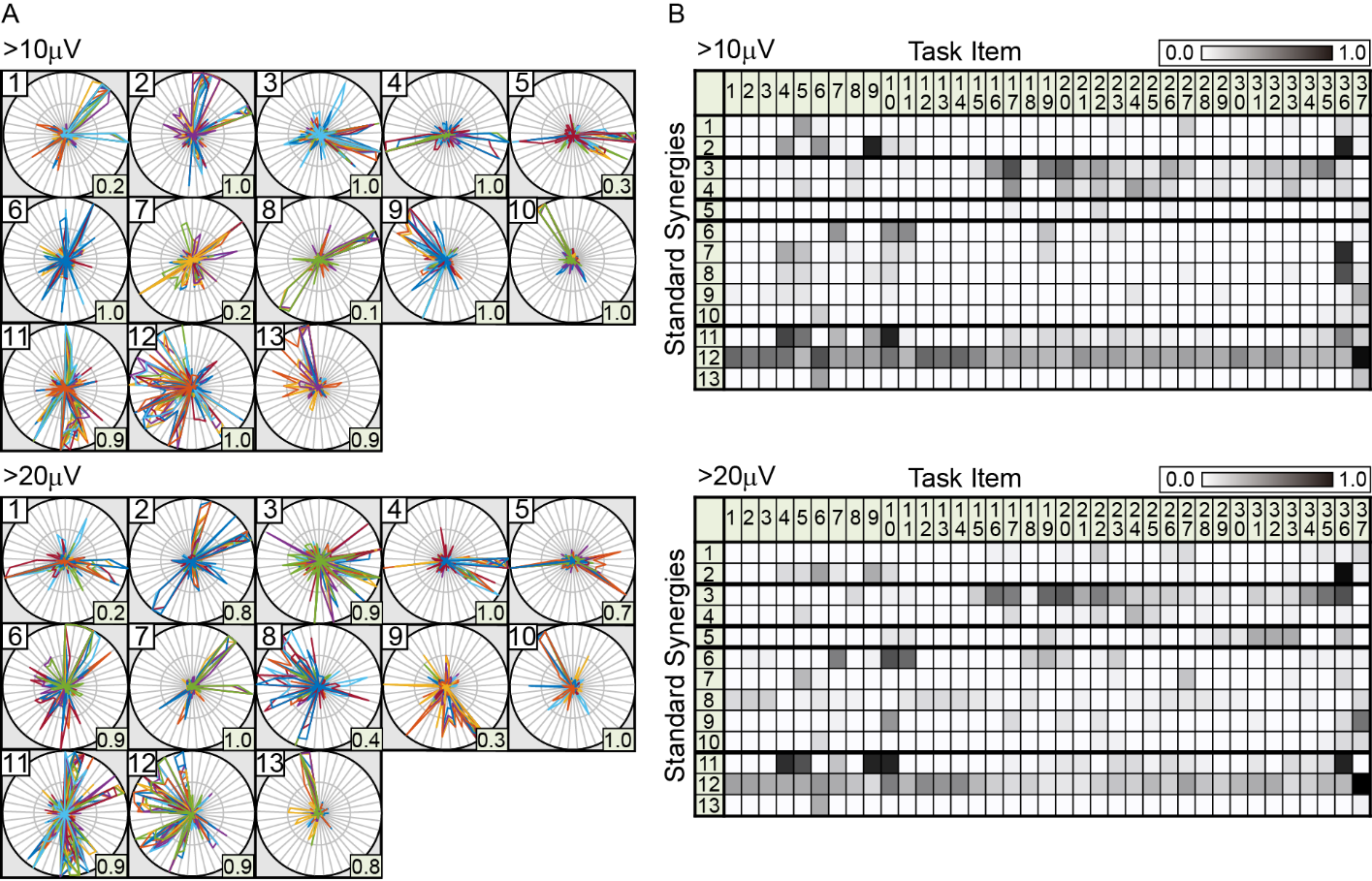


**Supplementary Figure 1: Standard synergies obtained by eliminating small EMG signals.** **(A)** Standard synergies. The numbers at the top left of each figure are the indices of the synergies and the numbers at the bottom right are the cosine correlations with the original standard synergies (Fig. 1B). Each color in the figure represents the standard synergies for each trial. To calculate the cosine correlation, the mean standard synergies for all trials were compared between the standard synergies with and without eliminating small EMG signals. **(B)** Standard synergy-task relationship. Synergies that were used in each task item are displayed in terms of the number of active trials normalized by the total number of trials in healthy participants.


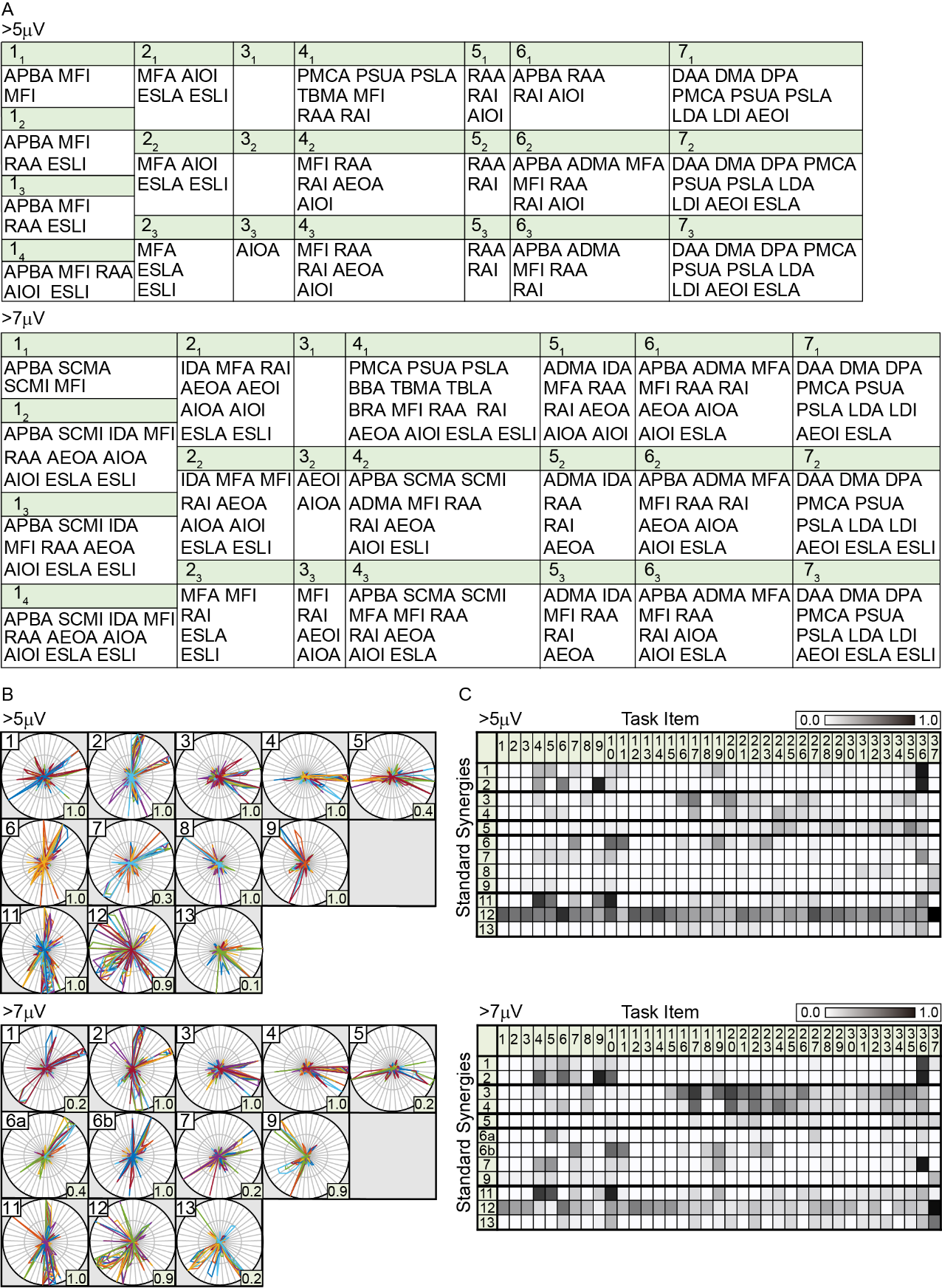


**Supplementary Figure 2: Standard synergies obtained by eliminating the muscles with small EMG signals.** **(A)** The muscles eliminated because EMG activity was small in each trial. The numbers represent the trial indices. **(B)** Standard synergies. The numbers at the top left of each figure represent the indices of the synergies and the numbers at the bottom right represent the cosine correlation with the original standard synergies (Fig. 1B). **(C)** Standard synergy-task relationship.


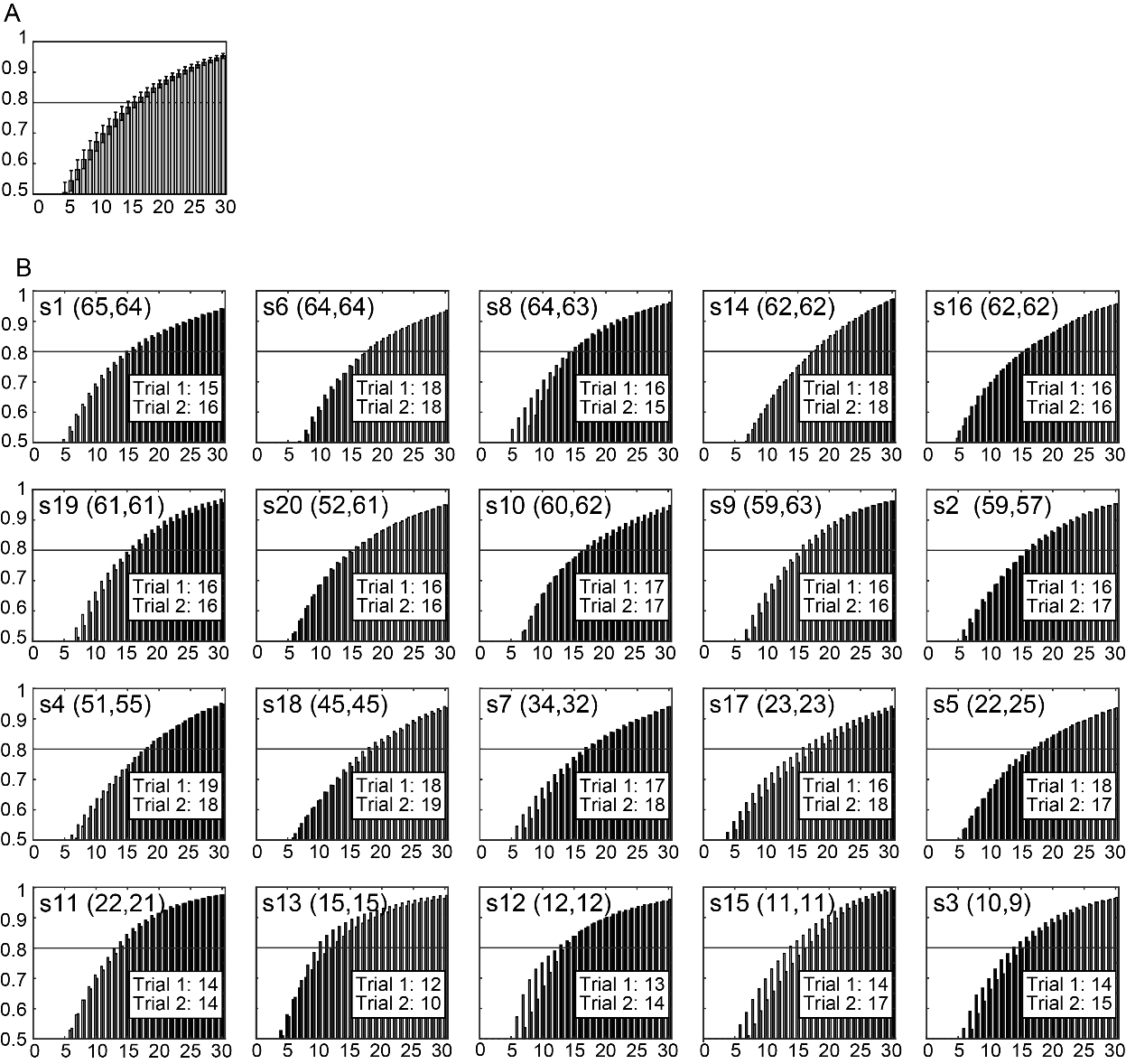


**Supplementary Figure 3: Variance accounted for (VAF) by muscle synergies.** (A) VAF in healthy participants. Each bar represents the average and standard deviation of the VAF for all trials and all participants. (B) VAF in patients with stroke. Each figure shows the results for one participant. Each bar in the figure shows the result from one trial. The numbers next to the IDs of the patients are the FMA scores. Data are arranged from the participant with the highest FMA score (top left) to the participant with the lowest FMA score (bottom right). The numbers in the white boxes are the minimum numbers of synergies that exceed VAF > 0.8.


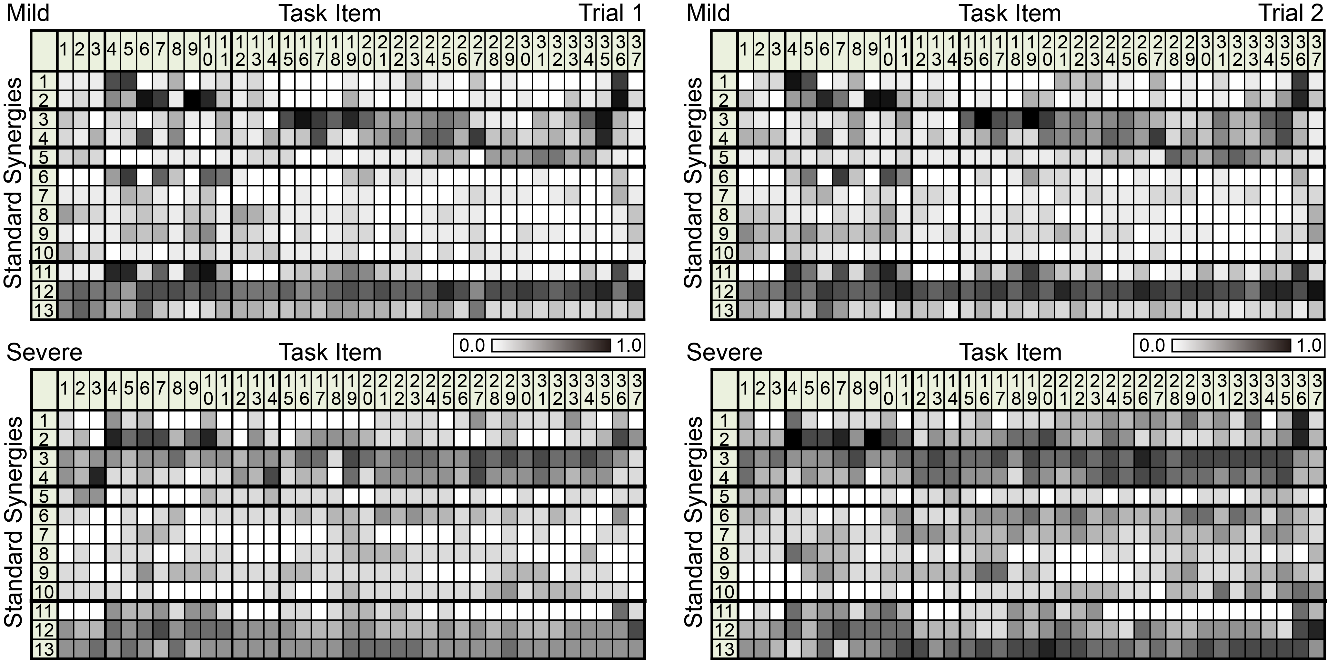


**Supplementary Figure 4: Standard synergy-task relationships of stroke patients.** Upper and lower panels show standard synergy-task relationships for participants who had experienced mild (FMA score > 30) and severe (FMA score < 30) stroke, respectively.


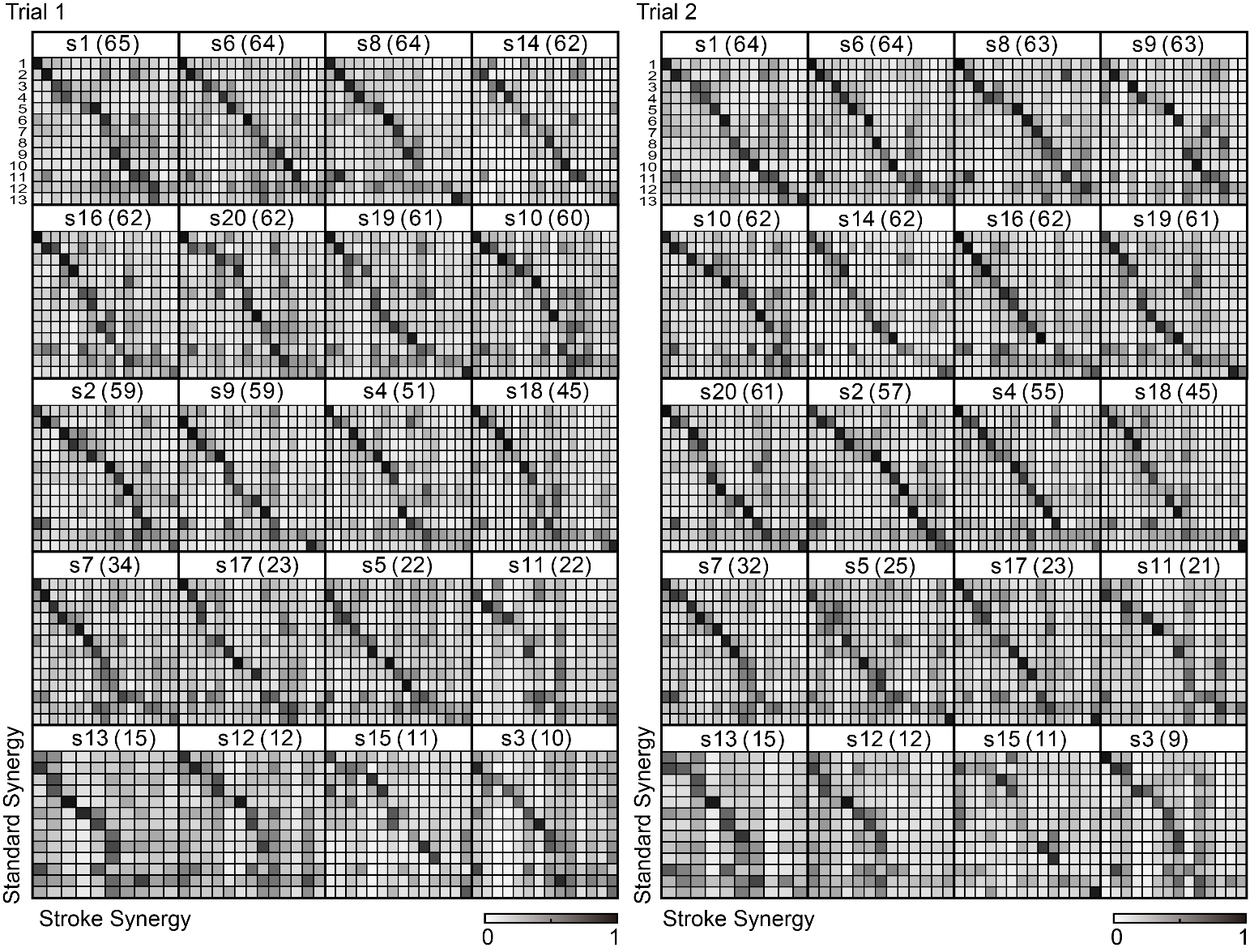


**Supplementary Figure 5: Correlation between standard synergies and stroke synergies.** Rows show the 13 standard synergies and columns show the stroke synergies. Shading indicates the value of the correlation coefficient between the row (standard synergy) and the column (stroke synergy). Black shaded sections indicate a high value and white indicates a low value. The ID of each stroke patient is shown on each figure. The numbers next to the IDs are the FMA scores. Data are arranged from the participant with the highest FMA score (top left) to the participant with the lowest FMA score (bottom right).


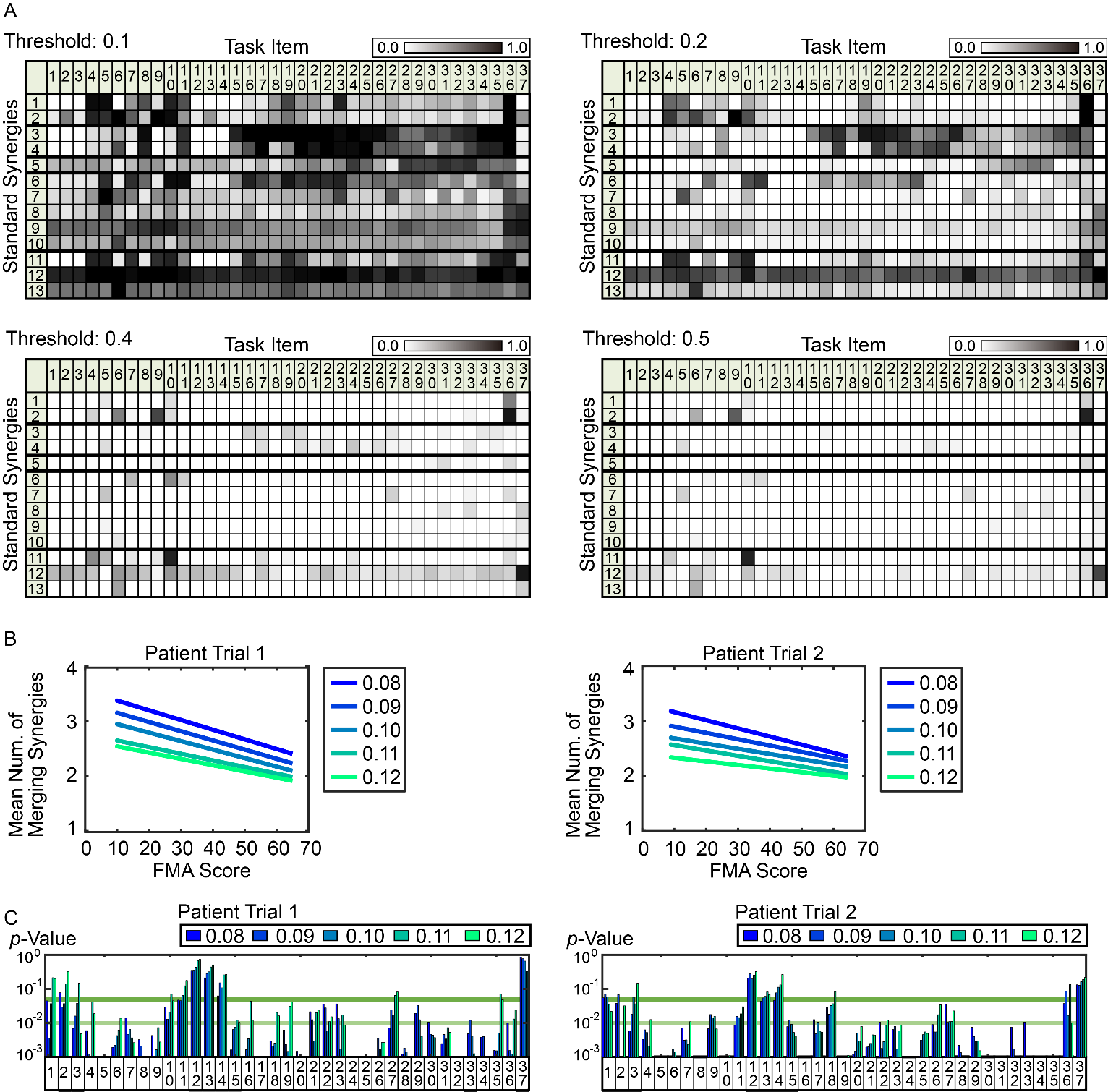


**Supplementary Figure 6: Effects of different thresholds for the synergy-task relationship and synergy merging.** (A) Synergy-task relationship with thresholds of 0.1, 0.2, 0.4, and 0.5. Similar to Fig. 2A, the synergies observed in each task item are displayed according to the number of active trials normalized by the total number of trials. (B) The merging rate for each stroke participant with different merging thresholds. Linear regression lines with different thresholds are displayed similar to Fig. 3B. (C) Task dependency of the severity-merging rate relationship with different merging thresholds. The *p*-values for the linear regression between severity and merging rate for each task are displayed similarly to those in Fig. 4B.


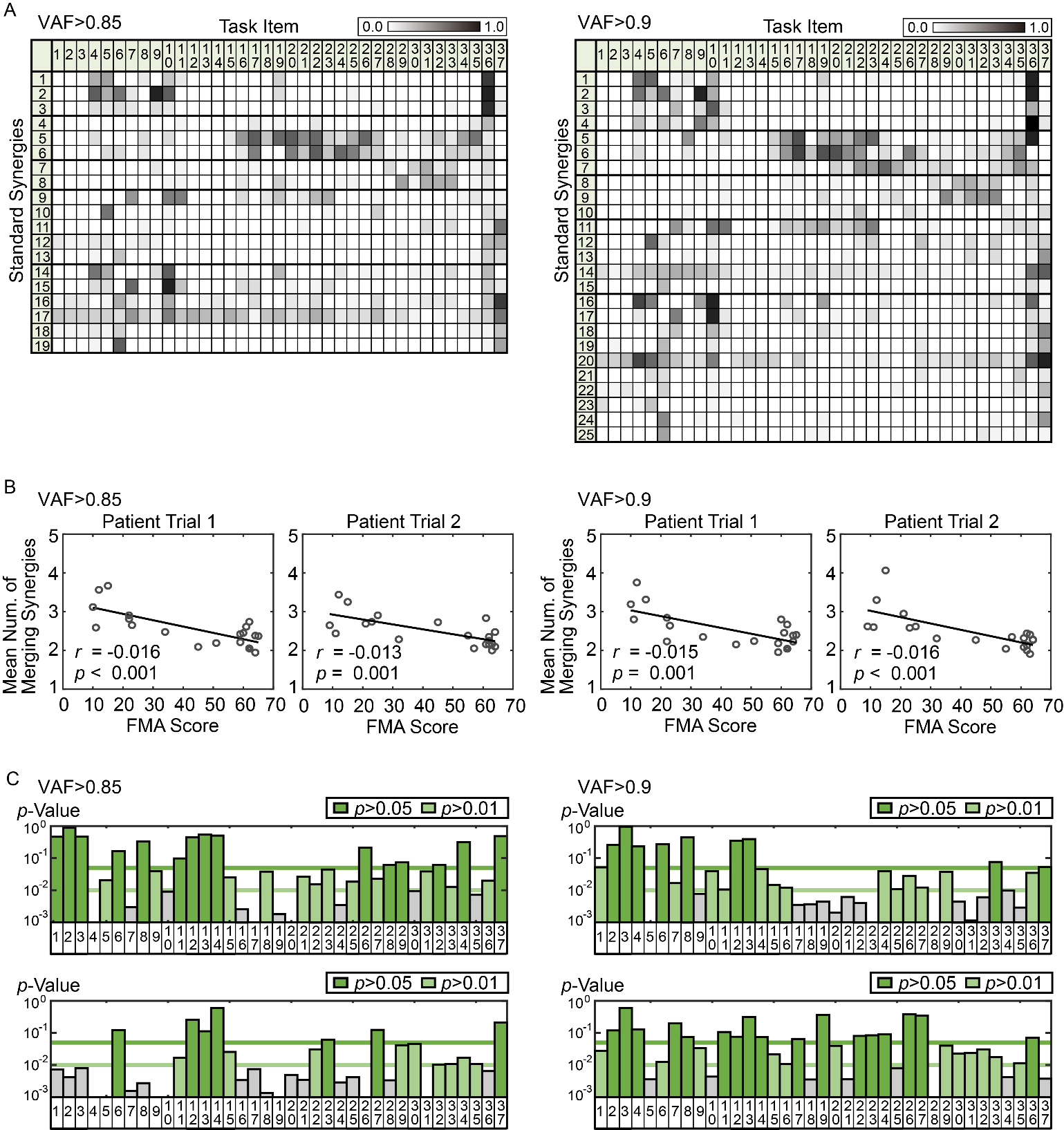


**Supplementary Figure 7: Effects of different VAF thresholds.** (A) Synergy-task relationship calculated with VAF thresholds of 0.85 and 0.9. Similar to Fig. 2A, the synergies observed in each task item are displayed according to the number of active trials normalized by the total number of trials. (B) The merging rate for each stroke participant with different VAF thresholds. Each point shows the merging rate for stroke patients in terms of FMA score (*n* = 20). Linear regression lines with different thresholds are displayed similarly to those in Fig. 3B. (C) Task dependency of the severity-merging rate relationship with different VAF thresholds. The *p*-values for the linear regression between severity and merging rate for each task are displayed similarly to those in Fig. 4B.

**Supplementary Table 1: Diagnostic and clinical data from patients with stroke.** The clinical data were evaluated by the therapist in charge of each patient on the closest possible date to the experimental assessment.

|  | Age (years) | Sex | Diagnosis | Type of  disability | Affected side | FIM-m | FIM-c | FMA upper extremity | FMA lower extremity | MMSE |
| --- | --- | --- | --- | --- | --- | --- | --- | --- | --- | --- |
| s1 | 49 | Male | Ischemic stroke  (bilateral ACA territory) | Hemiplegia | Left | 31 | 35 | 59 | 13 | 30 |
| s2 | 51 | Male | Brainstem hemorrhage  (medial to dorsal pons) | Hemiplegia | Left | 65 | 35 | 54 | 31 | 30 |
| s3 | 44 | Male | Right putaminal hemorrhage | Hemiplegia | Right | 65 | 32 | 9 | 14 | 30 |
| s4 | 68 | Male | Ischemic stroke  (left MCA territory) | Hemiplegia | Left | 79 | 35 | 51 | 25 | 30 |
| s5 | 34 | Male | Right subcortical hemorrhage  (right frontal to parietal lobe) | Hemiplegia | Right | 67 | 25 | 22 | 14 | 27 |
| s6 | 73 | Male | Ischemic stroke  (right posterior limb of the internal capsule–corona radiata) | Hemiplegia | Right | 72 | 35 | 64 | 28 | 30 |
| s7 | 69 | Male | Left thalamic hemorrhage | Hemiplegia | Left | 48 | 28 | 44 | 29 | 27 |
| s8 | 46 | Male | Left putaminal hemorrhage | Hemiplegia | Left | 78 | 30 | 62 | 32 | 30 |
| s9 | 44 | Male | Right putaminal hemorrhage | Hemiplegia | Right | 69 | 33 | 41 | 31 | 30 |
| s10 | 67 | Male | Ischemic stroke  (left corona radiata) | Hemiplegia | Left | 63 | 27 | 58 | 28 | 25 |
| s11 | 44 | Male | Right thalamic hemorrhage | Hemiplegia | Right | 56 | 29 | 14 | 13 | 30 |
| s12 | 64 | Male | Ischemic stroke  (left corona radiata) | Hemiplegia | Left | 74 | 33 | 13 | 20 | 30 |
| s13 | 72 | Male | Ischemic stroke  (right corona radiata) | Hemiplegia | Right | 44 | 34 | 6 | 16 | 25 |
| s14 | 37 | Male | Left putaminal hemorrhage | Hemiplegia | Right | 90 | 34 | 62 | 34 | 30 |
| s15 | 63 | Male | Ischemic stroke  (left corona radiata) | Hemiplegia | Right | 71 | 33 | 11 | 20 | 30 |
| s16 | 28 | Male | Right subcortical hemorrhage  (right frontal to parietal lobe) | Hemiplegia | Left | 86 | 35 | 62 | 12 | 30 |
| s17 | 63 | Male | Right thalamic hemorrhage | Hemiplegia | Left | 47 | 29 | 23 | 14 | 28 |
| s18 | 67 | Male | Ischemic stroke  (right medial medulla oblongata) | Hemiplegia | Left | 76 | 34 | 45 | 16 | 30 |
| s19 | 42 | Male | Right putaminal hemorrhage | Hemiplegia | Left | 50 | 31 | 61 | 14 | 30 |
| s20 | 65 | Male | Right thalamic hemorrhage | Hemiplegia | Left | 56 | 27 | 62 | 29 | 30 |

FIM-m: motor Functional Independence Measure

FIM-c: cognitive Functional Independence Measure

FMA upper extremity: Fugl-Meyer Assessment for upper extremity

FMA lower extremity: Fugl-Meyer Assessment for lower extremity

MMSE: Mini-Mental State Examination

ACA: anterior cerebral artery

MCA: middle cerebral artery

**Supplementary Table 2: Dates and locations of the experiments.** s1–s20 display data from stroke patients and h1–h7 display data from healthy participants.

|  | Date | Location |  |  | Date | Location |
| --- | --- | --- | --- | --- | --- | --- |
| s1 | Nov. 3, 2016 | Morinomiya Hospital, Osaka |  | h1 | Apr. 29, 2017 | The University of Tokyo, Hongo Campus |
| s2 | Nov. 3, 2016 | Morinomiya Hospital, Osaka |  | h2 | Apr. 29, 2017 | The University of Tokyo, Hongo Campus |
| s3 | Dec. 14, 2016 | Morinomiya Hospital, Osaka |  | h3 | May 8, 2017 | The University of Tokyo, Hongo Campus |
| s4 | Dec. 14, 2016 | Morinomiya Hospital, Osaka |  | h4 | May 22, 2017 | The University of Tokyo, Hongo Campus |
| s5 | Dec. 14, 2016 | Morinomiya Hospital, Osaka |  | h5 | June 5, 2017 | The University of Tokyo, Hongo Campus |
| s6 | Dec. 14, 2016 | Morinomiya Hospital, Osaka |  | h6 | June 12, 2017 | The University of Tokyo, Hongo Campus |
| s7 | June 23, 2017 | Morinomiya Hospital, Osaka |  | h7 | July 3, 2017 | The University of Tokyo, Hongo Campus |
| s8 | June 23, 2017 | Morinomiya Hospital, Osaka |  |  |  |  |
| s9 | June 23, 2017 | Morinomiya Hospital, Osaka |  |  |  |  |
| s10 | June 23, 2017 | Morinomiya Hospital, Osaka |  |  |  |  |
| s11 | Sept. 12, 2018 | Morinomiya Hospital, Osaka |  |  |  |  |
| s12 | Sept. 12, 2018 | Morinomiya Hospital, Osaka |  |  |  |  |
| s13 | Sept. 12, 2018 | Morinomiya Hospital, Osaka |  |  |  |  |
| s14 | Dec. 4, 2021 | Morinomiya Hospital, Osaka |  |  |  |  |
| s15 | Dec. 4, 2021 | Morinomiya Hospital, Osaka |  |  |  |  |
| s16 | Dec. 18, 2021 | Morinomiya Hospital, Osaka |  |  |  |  |
| s17 | Dec. 18, 2021 | Morinomiya Hospital, Osaka |  |  |  |  |
| s18 | Dec. 18, 2021 | Morinomiya Hospital, Osaka |  |  |  |  |
| s19 | Jan. 8, 2022 | Morinomiya Hospital, Osaka |  |  |  |  |
| s20 | Jan. 8, 2022 | Morinomiya Hospital, Osaka |  |  |  |  |

**Supplementary Table 3: List of Fugl-Meyer Assessment (FMA) tasks and the importance of each task.** The participants performed the 37 items sequentially in order from task 1 to task 37. Performance for each task was evaluated by medical professionals and given a score between 0 and 2. The total maximum score was 66.

| **Task**  **index** |  | **Motion of the task** | | |  | **Max.**  **score** |
| --- | --- | --- | --- | --- | --- | --- |
| 1, 3 |  | Reflex | 1: Biceps, 3: Finger | |  | 2 |
| 2 |  |  | Triceps |  |  | 2 |
| 4 |  | Flexor synergy | Forearm | Supination |  | 2 |
|  |  |  | Elbow | Flexion |  | 2 |
|  |  |  | Shoulder | Abduction |  | 2 |
|  |  |  |  | Outward rotation |  | 2 |
|  |  |  |  | Elevation |  | 2 |
|  |  |  |  | Retraction |  | 2 |
| 5 |  | Extensor synergy | Shoulder | Adduction/inward rotation |  | 2 |
|  |  |  | Elbow | Extension |  | 2 |
|  |  |  | Forearm | Pronation |  | 2 |
| 6 |  | Hand to lumbar spine | Hand | Move to lumbar spine |  | 2 |
| 7 |  |  | Shoulder | 0°–90° |  | 2 |
| 8 |  |  | Elbow 90° | Pronation/supination |  | 2 |
| 9 |  | Shoulder | Abduction 0°–90° | |  | 2 |
| 10 |  |  | Flexion 90°–180° | |  | 2 |
| 11 |  | Elbow 0° | Pronation/Supination | |  | 2 |
| 12, 13, 14 |  | Normal reflex | 12: Biceps, 13: Finger, 14: Triceps | |  | 2 |
| 15, 16 |  | Wrist | Elbow 90° | Wrist stability  (16: with resistance) |  | 2 |
| 17 |  |  |  | Wrist flexion/extension |  | 2 |
| 18, 19 |  |  | Elbow 0° | Wrist stability  (19: with resistance) |  | 2 |
| 20 |  |  |  | Wrist flexion/extension |  | 2 |
| 21, 22, 23 |  | Circumduction | 21: Supination, 22: Pronation, 3: Pronation/supination | |  | 2 |
| 24 |  | Hand | Fingers mass flexion | |  | 2 |
| 25 |  |  | Fingers mass extension | |  | 2 |
| 26, 27 |  |  | Grasp | Pip-dip-hook (27: with resistance) |  | 2 |
| 28, 29 |  |  |  | Lateral-pinch (29: with resistance) |  | 2 |
| 30, 31 |  |  |  | Pulp-pinch (31: with resistance) |  | 2 |
| 32, 33 |  |  |  | Cylinder grasp (33: with resistance) |  | 2 |
| 34, 35 |  |  |  | Spherical grasp (35: with resistance) |  | 2 |
| 36, 37 |  | Coordination  /speed in finger–nose test | Tremor | (36: affected side, 37: unaffected side) |  | 2 |
|  |  |  | Dysmetria | (36: affected side, 37: unaffected side) |  | 2 |
|  |  |  | Speed | (36: affected side, 37: unaffected side) |  | 2 |
|  |  |  |  | Total score |  | 66 |

**Supplementary Table 4: List of measured muscles.** ‘Ch’ refers to the sensor channel. ‘Abbrev.’ is an abbreviation of the muscle name combined with the side that was measured.

| Ch | Name of muscle | Measured side | Abbrev. | |
| --- | --- | --- | --- | --- |
| 1 | Deltoideus, anterior | Affected | | DAA |
| 2 | Deltoideus, middle | Affected | | DMA |
| 3 | Deltoideus, posterior | Affected | | DPA |
| 4 | Pectoralis major, clavicular part | Affected | | PMCA |
| 5 | Pectoralis major, sternocostal, upper | Affected | | PSUA |
| 6 | Pectoralis major, sternocostal, lower | Affected | | PSLA |
| 7 | Biceps brachii | Affected | | BBA |
| 8 | Triceps brachii, medialis | Affected | | TBMA |
| 9 | Triceps brachii, lateralis | Affected | | TBLA |
| 10 | Brachioradialis | Affected | | BRA |
| 11 | Flexor carpi radialis | Affected | | FCRA |
| 12 | Flexor carpi ulnaris | Affected | | FCUA |
| 13 | Extensor carpi radialis | Affected | | ECRA |
| 14 | Extensor carpi ulnaris | Affected | | ECUA |
| 15 | Abductor pollicis brevis | Affected | | APBA |
| 16 | Pronator teres | Affected | | PTA |
| 17 | Sternocleidomastoid | Affected | | SCMA |
| 18 | Sternocleidomastoid | Intact | | SCMI |
| 19 | Trapezius, upper | Affected | | TUA |
| 20 | Trapezius, middle | Affected | | TMA |
| 21 | Trapezius, lower | Affected | | TLA |
| 22 | Serratus anterior | Affected | | SAA |
| 23 | Latissimus dorsi | Affected | | LDA |
| 24 | Latissimus dorsi | Intact | | LDI |
| 25 | Infraspinatus | Affected | | ISA |
| 26 | Erector spinae, cervical part | Affected | | ESCA |
| 27 | Erector spinae, cervical part | Intact | | ESCI |
| 28 | Erector spinae, thoracic part | Affected | | ESTA |
| 29 | Erector spinae, thoracic part | Intact | | ESTI |
| 30 | Abductor digiti minimi | Affected | | ADMA |
| 31 | Interossei dorsales | Affected | | IDA |
| 32 | Multifidus | Affected | | MFA |
| 33 | Multifidus | Intact | | MFI |
| 34 | Rectus abdominis | Affected | | RAA |
| 35 | Rectus abdominis | Intact | | RAI |
| 36 | Abdominal external oblique | Affected | | AEOA |
| 37 | Abdominal external oblique | Intact | | AEOI |
| 38 | Abdominal internal oblique | Affected | | AIOA |
| 39 | Abdominal internal oblique | Intact | | AIOI |
| 40 | Erector spinae, lumbar part | Affected | | ESLA |
| 41 | Erector spinae, lumbar part | Intact | | ESLI |

**Supplementary Table 5: The muscles and body regions that correspond to each standard synergy.** ‘Synergy’ indicates the index of the standard synergy. ‘Muscle’ refers to the main muscles activated in each standard synergy. ‘Area’ is the body region in which the muscles are primarily located. Muscles with white backgrounds were found using thresholds > 0.6, those with light gray were found using thresholds > 0.5, and those with dark gray were found using thresholds > 0.4.

| **Synergy** | **Muscles** | **Area** |
| --- | --- | --- |
| **1** | BBA | Upper arm |
| **2** | DAA | Upper arm |
|  | DMA |  |
|  | DPA |  |
|  | TBMA |  |
|  | TBLA |  |
| **3** | BRA | Forearm |
|  | ECRA |  |
| **4** | FCRA | Forearm |
|  | FCUA |  |
| **5** | APBA | Finger |
| **6** | PMCA | Chest |
| **7** | PSUA | Chest |
|  | PSLA |  |
| **8** | AEOA | Abdomen |
| **9** | AEOI | Abdomen |
|  | AIOA |  |
| **10** | AIOI | Abdomen |
| **11** | DAA | Posterior trunk (upper) |
|  | DMA |  |
|  | TUA |  |
|  | TMA |  |
|  | TLA |  |
|  | SAA |  |
|  | ISA |  |
| **12** | APBA | Posterior trunk (middle) |
|  | SCMA |  |
|  | SCMI |  |
|  | LDI |  |
|  | ESCA |  |
|  | ESCI |  |
|  | ESTA |  |
|  | ESTI |  |
|  | MFA |  |
|  | MFI |  |
|  | RAI |  |
| **13** | ESLA | Posterior trunk (lower) |
|  | ESLI |  |

All muscle abbreviations are listed in Supplementary Table 4.

**Supplementary Table 6: List of removed subject data to satisfy the assumption of linear regression in the merging-severity relationship for tasks.** No subject data were removed in the tasks that are not listed here.

Trial 1

| Task number | 6 | 8 | 11 | 15 | 17 | 21 | 24 | 34 | 35 | 36 |
| --- | --- | --- | --- | --- | --- | --- | --- | --- | --- | --- |
| Removed subject(s) | s11 | s8 | s9 | s9 | s9 | s8 | s12 | s2 | s6 | s3 |
|  |  | s10 |  |  |  | s9 |  | s11 |  |  |

Trial 2

| Task number | 5 | 6 | 8 | 17 | 19 | 29 | 24 | 31 | 34 | 36 |
| --- | --- | --- | --- | --- | --- | --- | --- | --- | --- | --- |
| Removed subject(s) | s5 | s7 | s12 | s6 | s7 | s3 | s12 | s5 | s7 | s11 |
|  | s20 | s16 |  | s13 |  |  |  | s7 |  |  |

# Supplementary References

1. Lee DD, Seung HS. Learning the parts of objects by non-negative matrix factorization. *Nature*. 1999; 401: 788-91.

2. Tresch MC, Cheung VC, d'Avella A. Matrix factorization algorithms for the identification of muscle synergies: evaluation on simulated and experimental data sets. *J Neurophysiol*. 2006; 95: 2199-212.

3. Turpin NA, Uriac S, Dalleau G. How to improve the muscle synergy analysis methodology? *Eur J Appl Physiol*. 2021; 121: 1009-25.

4. Cheung VC, Turolla A, Agostini M, et al. Muscle synergy patterns as physiological markers of motor cortical damage. *Proc Natl Acad Sci U S A*. 2012; 109: 14652-6.
